# A globally consistent local-scale assessment of future tropical cyclone risk

**DOI:** 10.1126/sciadv.abm8438

**Published:** 2022-04-27

**Authors:** Nadia Bloemendaal, Hans de Moel, Andrew B. Martinez, Sanne Muis, Ivan D. Haigh, Karin van der Wiel, Reindert J. Haarsma, Philip J. Ward, Malcolm J. Roberts, Job C. M. Dullaart, Jeroen C. J. H. Aerts

**Affiliations:** 1 Institute for Environmental Studies (IVM), Vrije Universiteit Amsterdam, 1081 HV Amsterdam, Netherlands.; 2 Office of Macroeconomic Analysis, U.S. Department of the Treasury, 1500 Pennsylvania Ave., NW, Washington, DC 20220, USA.; 3 Climate Econometrics, Nuffield College, Oxford OX1 1NF, UK.; 4 Deltares, 2600 MH Delft, Netherlands.; 5 School of Ocean and Earth Science, National Oceanography Centre, University of Southampton, European Way, Southampton SO14 3ZH, UK.; 6 Royal Netherlands Meteorological Institute (KNMI), 3731 GA De Bilt, Netherlands.; 7 Met Office, Exeter, UK.

## Abstract

There is considerable uncertainty surrounding future changes in tropical cyclone (TC) frequency and intensity, particularly at local scales. This uncertainty complicates risk assessments and implementation of risk mitigation strategies. We present a novel approach to overcome this problem, using the statistical model STORM to generate 10,000 years of synthetic TCs under past (1980–2017) and future climate (SSP585; 2015–2050) conditions from an ensemble of four high-resolution climate models. We then derive high-resolution (10-km) wind speed return period maps up to 1000 years to assess local-scale changes in wind speed probabilities. Our results indicate that the probability of intense TCs, on average, more than doubles in all regions except for the Bay of Bengal and the Gulf of Mexico. Our unique and innovative methodology enables globally consistent comparison of TC risk in both time and space and can be easily adapted to accommodate alternative climate scenarios and time periods.

## Teaser

A recently developed synthetic model predicts local-scale changes in assessments of future global tropical cyclone risk.

## INTRODUCTION

Tropical cyclones (TCs) are responsible for the highest insured losses of any natural hazard, exceeding $480 billion in the United States alone over the last decade (*1*). TCs are projected to become more intense in a warming climate (*2*), enhancing the risks associated with their wind speeds, precipitation, storm surges, and waves (*3*, *4*). TC losses have shown to rise steeply and nonlinearly with increasing intensity (*5*–*9*). Moreover, TC losses can vary greatly across spatial scales, with the largest losses often found in more densely populated areas. Hence, to better protect coastal communities from future TC impacts, it is vital to improve our understanding of future changes in local-scale TC hazards and risk.

Future climate TC impact assessments often rely on climate projections from global climate models (GCMs) for specific scenarios of carbon emissions. However, GCMs provide limited information on how climate extremes such as TCs may change (*10*, *11*), primarily because the spatial resolution of past-generation GCMs (±1.0°) is insufficient to adequately resolve TC intensity, size, and track (*12*). Consequently, there is no consensus on the projected change in TC frequency and characteristics under various climate change scenarios (*2*), particularly at the local scale. Recently, substantial progress has been made with the development of high-resolution GCMs (±0.25°) (*13*). However, these GCMs still struggle to capture the most intense TCs (*10*), both through continued limitations in resolution and numerical precision (*14*) and from parameterizations of convective processes that do not hold for intense TCs, such as the assumption of hydrostatic balance. In addition, GCM simulations typically only cover 30- to 100-year periods of historical and future climate (*13*), resulting in a small sample of TCs. This is an important constraint that further limits the accurate estimation of (changes in) the probability of extreme events in time and in space.

Several approaches exist to overcome the issues of poor TC representation and short simulations. One method to simulate future-climate TCs in a high-resolution setting is the pseudo–global warming (PGW) approach. Such experiments consist of a reference experiment forced with baseline climate conditions, and a “future” experiment in which a systematic change is added to these baseline conditions. PGW experiments are specifically designed to simulate a specific weather event (such as a historical TC) in a different climate setting while keeping the meteorological dynamical details of the event the same (*15*–*17*). Hence, a PGW experiment cannot be used to simulate new TCs that show unprecedented behavior, independent of that observed in the input dataset, or to investigate changes in probability. Another approach that does support the creation of new TCs is the so-called dynamical downscaling approach (*5*, *18*). In this approach, atmospheric variables are extracted from GCMs to create a large-scale environment in which TCs are randomly seeded and simulated using a TC model. This downscaling approach, however, requires a substantial number of inputs, and the simulated effects of climate change on TCs are only caused by processes in the large-scale environment (e.g., changes in wind shear or enhanced atmospheric stability), rather than changes in, for instance, genesis frequency.

Another approach is synthetic modeling (*19*–*21*). Here, TC characteristics are extracted from either historical data (*21*, *22*) or GCM simulations (*4*), and are either statistically or dynamically resampled and modeled to generate a synthetic TC dataset, spanning thousands of years. This supports robust estimations of return periods (RPs) exceeding the temporal length of the input data. Such global-scale RP estimations were derived for the present climate by Bloemendaal *et al*. (*23*) and Lee *et al*. (*20*). In their future-climate study, Lee *et al*. (*18*) also present RP maps for category 4 TC events; however, these RPs were derived at 3.6° × 1.8° (±400 km × 200 km) grid cells, which is too coarse for local-scale TC risk assessments. Future-climate, global-scale, high-resolution RP maps for various TC wind speeds have not been derived thus far.

Here, we use the Synthetic Tropical cyclOne geneRation Model (STORM) (*19*) to create globally consistent wind speed RP maps at 10-km resolution and for various RPs up to 1000 years. To derive these hazard maps, we first design a framework to generate future-climate synthetic TCs, thereby combining the benefits of high-resolution GCMs and synthetic modeling (see Materials and Methods). In this framework, we extract information on changes in TC variables (1979–2014 versus 2015–2050) from four high-resolution GCM simulations based on a high-emission scenario. Next, we project these changes onto the same TC variables from historical data (*24*), which served as input for the STORM baseline climate dataset (STORM-B) (*19*). We thereby create input variables related to the future climate for each GCM. Next, we use this as input for STORM to simulate 10,000 years of future TC activity under climate change (STORM-C). Last, we convert the synthetic tracks to a two-dimensional (2D) wind field using a parametric model (*25*) and calculate the wind speed RPs at a 10-km resolution. All datasets presented in this study are publicly available and can be used as (forward looking) hazard maps in catastrophe models commonly used by the public (e.g., academia, policymakers, and nongovernmental organizations) and the private sectors [e.g., consultancy and (re)insurance companies].

## RESULTS

### Generation of synthetic tropical cyclones under climate change

**Fig. 1.**
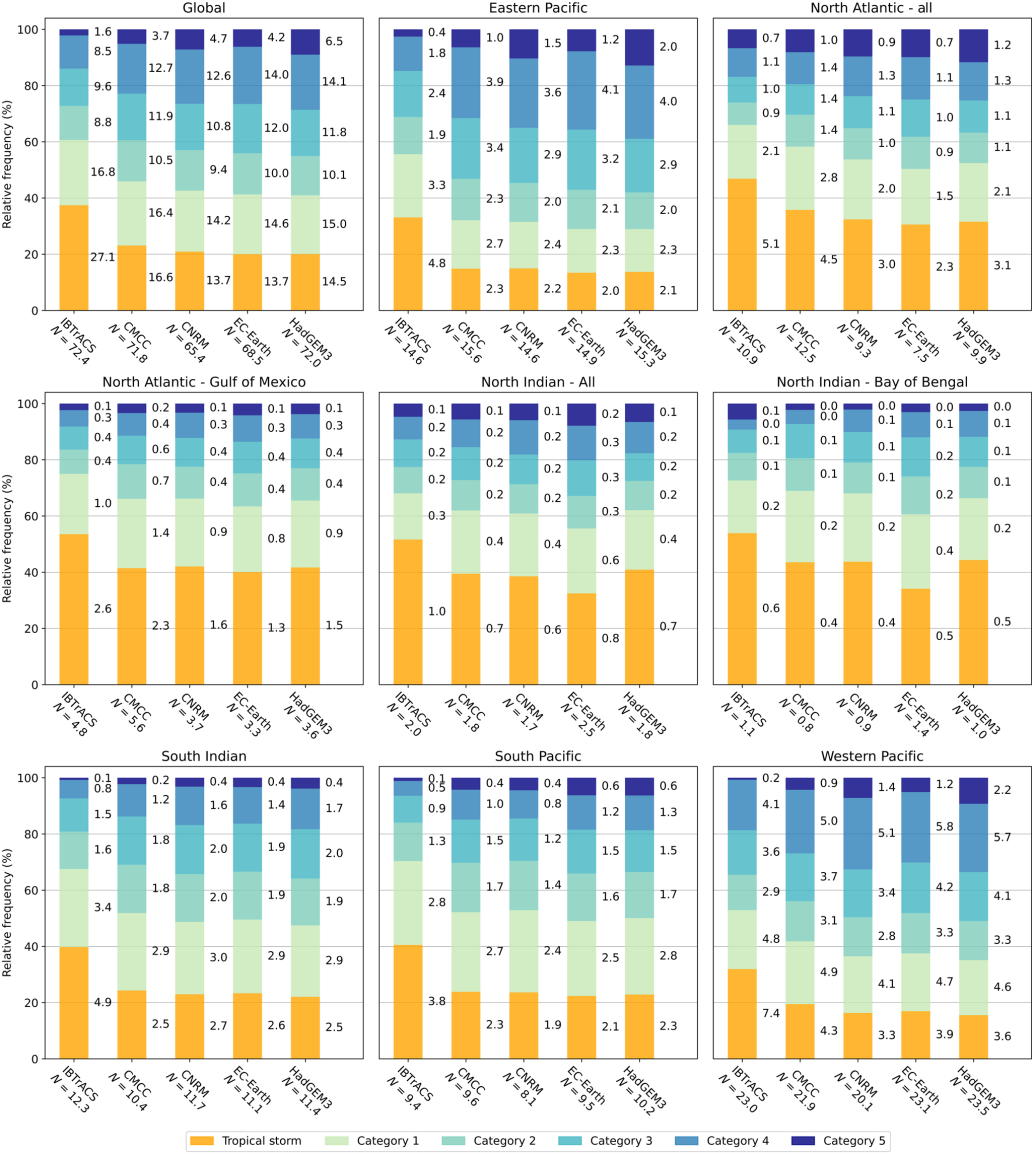
Relative and absolute frequency of different TC categories (tropical storm, category 1 to category 5) for 10,000 years of baseline climate STORM-B data and 10,000 years of future-climate STORM-C data for each of the four GCMs. Relative numbers are indicated by the height of the colored bar chart; absolute frequency (given as average per year) per TC category and per input dataset are indicated to the right of the respective colored bar chart. The total number of cyclone formations is indicated by *N* in the *x* axis label. We plot the global statistics, the six basins originally modeled by STORM (Eastern Pacific, North Atlantic, North Indian, South Indian, South Pacific, and Western Pacific), and two subregions, the Gulf of Mexico and the Bay of Bengal.

Our results show a global-scale increase in the frequency of occurrence of intense TCs (i.e., exceeding category 3 on the Saffir-Simpson Hurricane Wind Scale) (*26*) and a decrease in the frequency of weaker systems such as tropical storms (Fig. 1). We find similar results for the different basins except the Bay of Bengal (North Indian), where the frequency of intense TCs decreases under climate change. This is in line with results from other studies using high-resolution (finer than 28 km × 28 km) GCMs (*2*)).

The results also indicate an increase in the magnitude of TC intensity, here assessed through the average maximum wind speed (meters per second) across all four STORM-C datasets (table S1). On a global scale, maximum wind speeds increase from 35.0 ± 1.9 m/s in the baseline (leftmost column in table S1) to an average of 39.9 ± 3.0 m/s across the future-climate STORM-C datasets (rightmost column in table S1), with all GCMs agreeing on the direction of change. In line with results obtained in other studies (*2*, *27*, *28*), our results also show a robust change on a basin scale. The largest differences in average maximum wind speeds are found in the Eastern Pacific, with an increase of 7.7 m/s compared to the baseline (37.0 to 44.7 m/s). Relative increases in maximum wind speeds between the baseline and the different future-climate datasets for the different basins lie between 7.2 and 23.8% (table S2). While the sign of change is the same, our range is higher than that of Knutson *et al*. (*2*), who report a range of 1 to 10% derived from synthesizing the results from many GCM-based studies. Possible reasons for these differences in range include (i) the strong dependency of TC intensity on sea surface temperatures (SSTs) in STORM. We calculate the maximum potential intensity (MPI; in hectopascal) from these SSTs, which serves as an environmental constraint on the maximum TC intensity. Regions with lower SSTs are therefore more susceptible to TC weakening. In reality, TC weakening is also governed by (among other factors) enhanced (vertical) wind shear, the entrainment of dry air, and the influence of nearby land masses (*29*, *30*). STORM does not simulate these processes, and therefore, TC weakening may be better represented in GCMs. (ii) Another potential cause for the difference in estimated intensity changes is the fact that STORM uses a lower bound for the maximum wind speed, set at 18 m/s. It is plausible that previous studies (*2*) did not contain such threshold and that the intensity increases under climate change are therefore somewhat tempered by these weaker storms. (iii) Last, the analysis by Knutson *et al*. (*2*) also consists of lower-resolution GCMs than used in our study (see Materials and Methods). Because TC intensity is commonly inadequately resolved in coarser-resolution GCMs (*12*), this might also drive the differences in the ranges of future maximum wind speed changes.

Besides the overall projected increase in TC intensity, the STORM-C datasets also show a robust increase in the frequency of intense (category 4 and category 5) TCs, with results ranging between 0.5 and 219% across the different regions (table S2). This increase is driven by the combination of an increase in TC intensity and a decrease in TC frequency (see below). Our range of increase falls well within the range presented by Knutson *et al*. (*2*), who deduced relative changes ranging between approximately −80 and 697% [see Figure 2 in Knutson *et al*. (*2*)]. For some regions, however, our estimates lie north of the 90th percentile value. This is particularly evident in the Southern Pacific, where our relative changes range between 97 and 218%, whereas the 90th percentile value of Knutson *et al*. (*2*) lies closer to 25%. However, they also show that the lower-resolution models are typically projecting a decrease in the frequency of intense TCs. This may skew the results toward lower relative changes compared to solely using high-resolution models such as those used in our study.

In agreement with literature (*2*), all four STORM-C datasets indicate a global decrease in annual TC genesis frequency compared to the baseline STORM-B dataset (table S1), amounting to 72.4 ± 1.4 in the baseline climate to an average of 69.6 ± 3.1 across the four future-climate datasets. For two of the four GCMs (CMCC and HadGEM3), the decrease is within 1 standard deviation (SD) of the baseline (72.0 ± 1.5 and 72.2 ± 1.5, respectively), and we therefore do not consider it to be statistically significant. At the basin level, however, the four STORM-C datasets only agree on the sign of change in the South Indian basin, while in the other regions, they do not.

The STORM-C datasets show a future poleward expansion of the location of maximum intensity in the Northern Hemisphere, particularly in the Western Pacific and the North Atlantic (fig. S1). This poleward shift is driven by an increase in SSTs at higher latitudes, supporting TC tracks further northward, and is consistent with previous studies (*31*, *32*).

### Changes in low-probability events

**Fig. 2.**
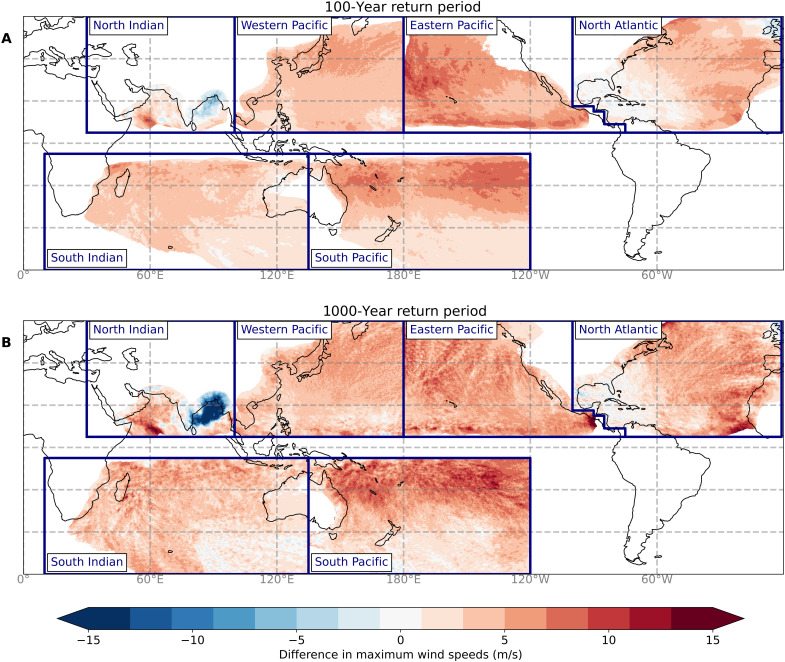
Change in 10-min 10-m average maximum wind speed between STORM-B and the ensemble median of the STORM-C datasets. The STORM-B and STORM-C datasets correspond to the average climate conditions of 1980-2017 and 2015-2050, respectively. Subpanels show the change in wind speed at the 100 (**A**) and 1000 (**B**) RPs, respectively. Red tones indicate a positive change (i.e., an increase in wind speed), and blue tones indicate a negative change.

The main advantage of our approach is that the large sample of synthetic TC tracks allows for a more robust assessment of changes in low-probability events. For this assessment, we derive wind speed RPs at high spatial resolution (10 km) by coupling the STORM-B and STORM-C datasets to a 2D wind parametric wind field (see Materials and Methods). For the ensemble median of the STORM-C datasets, we observe an increase in maximum wind speeds compared to STORM-B at most locations for the 100- and 1000-year RP (Fig. 2). The largest basin-scale mean increase is found in the Eastern Pacific, amounting to an average basin-scale increase of 5.5 m/s across all 10-km grid cells at the 1000-year RP (see tables S6 and S7). There are also regions for which our results show a decrease in maximum wind speeds such as the Bay of Bengal, where maximum wind speeds decrease up to 15 m/s for the 1000-year RP. This decrease is primarily driven by a shift in simulated TC genesis locations closer to the Indian/Sri Lankan land masses, associated with a northward shift in the Intertropical Convergence Zone (*33*). This shift increases the chances of a TC making landfall before intensifying further, predominantly reducing the formation chances of category 5 TCs (see also Fig. 1).

There is only a minor change in maximum wind speeds for both RPs in the Gulf of Mexico and the Caribbean, although our results do show an increase in the relative frequency of intense TCs in this region (Fig. 1). In this region, the absolute frequency of high-intensity TCs does not change much toward the future (2767 TCs in the baseline versus an ensemble median of 2624 across the future-climate datasets). However, as the total frequency of TCs decreases (seen in three of the four GCMs; see table S1), the relative frequency of intense TCs also decreases slightly, resulting in similar RP for these events. These findings are consistent with other literature (*34*). Note that STORM does not model extratropical transition of TCs; hence, maximum wind speeds and corresponding RP analyses in extratropical regions (poleward of 40°N/S) should be disregarded as they may be represented incorrectly. Furthermore, this RP analysis is carried out at the basin scale; hence, there is often no smooth transition of maximum wind speeds near the basin boundaries—this is, for instance, visible at the Eastern/Western Pacific basin boundary.

**Fig. 3.**
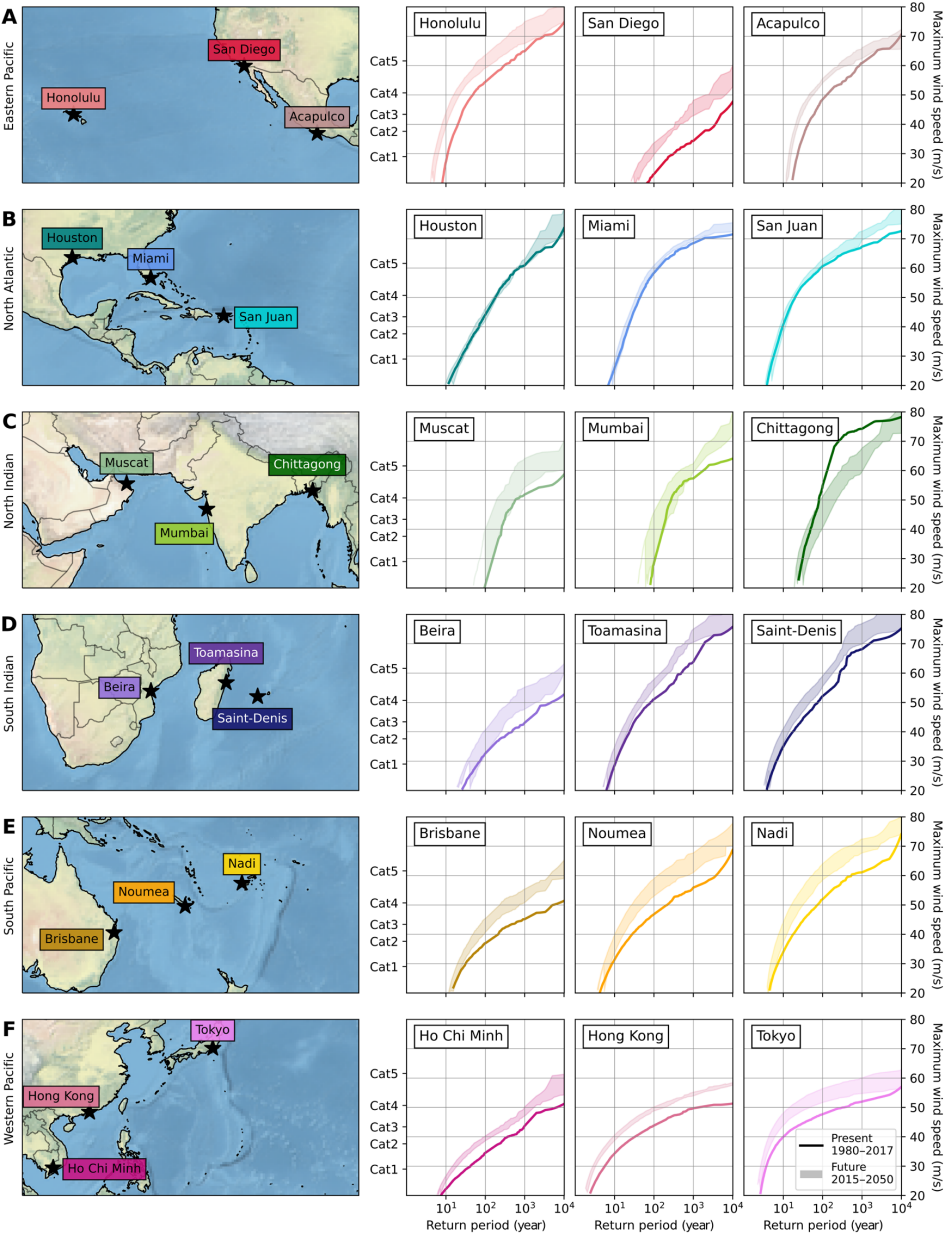
Empirically derived RPs of of maximum 10-min 10-m average wind speeds within a radius of 100 km for a selection of 18 coastal cities. The 18 coastal cities are evenly distributed over the six ocean basins: Eastern Pacific (**A**), North Atlantic (**B**), North Indian (**C**), South Indian (**D**), South Pacific (**E**), and Western Pacific (**F**). The solid line represents the STORM-B RPs (corresponding to the average climate conditions of 1980-2017). The shaded areas indicate the range of RPs of the four STORM-C datasets (corresponding to the average climate conditions of 2015-2050).

Next, we assess how RPs of maximum wind speeds change for 18 coastal megacities located in TC-prone regions (Fig. 3). For 14 cities, the simulations show an increase in maximum wind speeds across the range of RPs, indicating an increase in TC hazard. For Nouméa (New Caledonia), the maximum wind speed at the 100-year RP increases from 46.8 m/s in the baseline climate to 53.0 to 59.0 m/s under climate change, which is the largest increase across the 18 cities. At the 1000-year RP, the largest increase in maximum wind speed is found for San Diego (USA), increasing from 34.3 m/s to 42.9 to 48.2 m/s. The cities in the North Atlantic basin all lie within the Gulf of Mexico and Caribbean region, where the STORM-C dataset shows a very minor change in wind speed RPs. Hence, the STORM-C RP curves for these cities (Houston, Miami, and San Juan) deviate little from the STORM-B RP curve. To illustrate, the largest absolute change in the 100-year wind speed across these three cities is only 2.5 m/s for San Juan. For Chittagong (Bangladesh), located in the Bay of Bengal, the STORM-C RP curve is substantially lower than the baseline, especially for RPs ranging from 100 to 10,000 years.

**Fig. 4.**
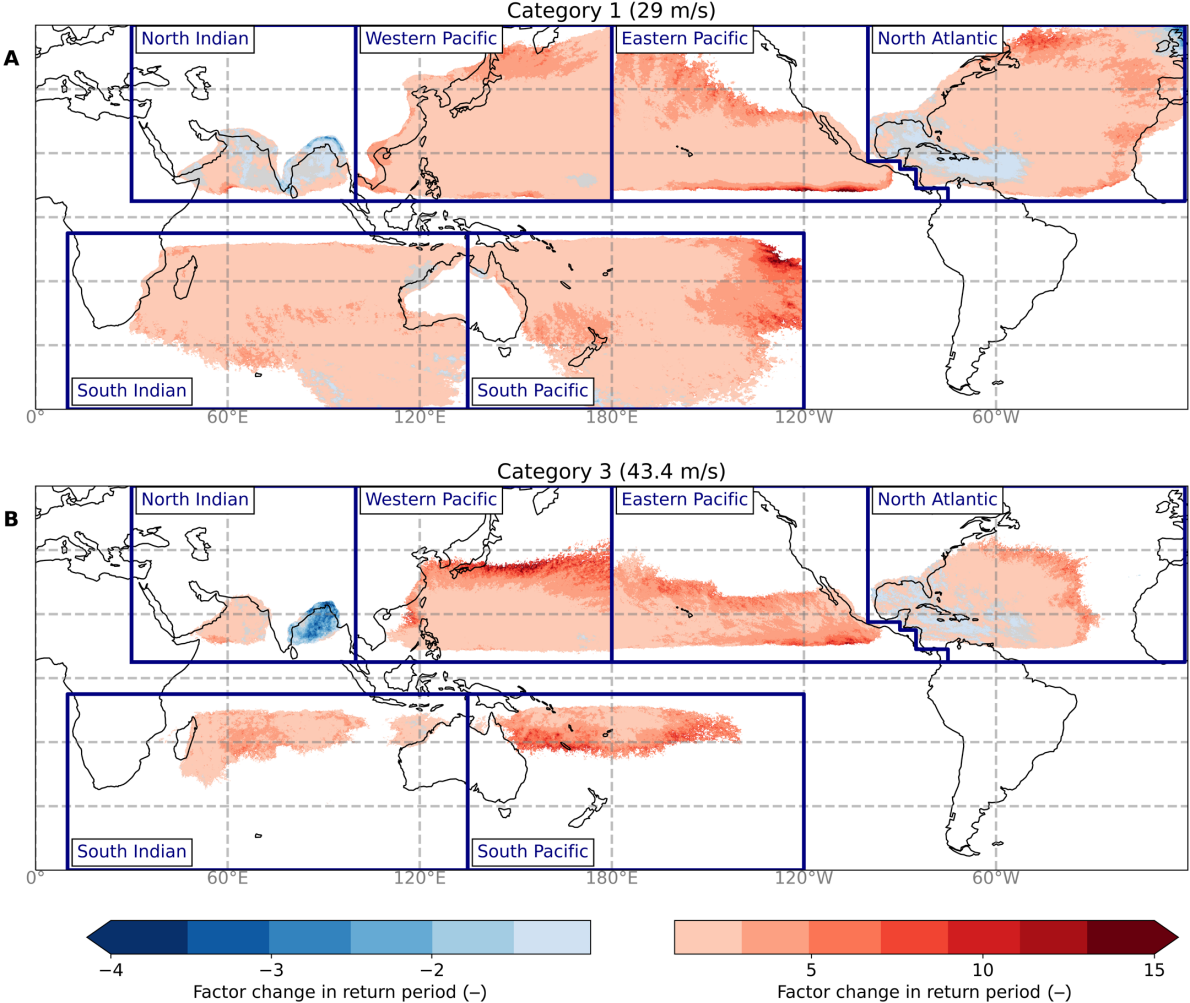
Ensemble median of the factor change in RP between the STORM-B and the STORM-C datasets for fixed 10-min 10-m wind speeds. TThe STORM-B and STORM-C datasets correspond to the average climate conditions of 1980-2017 and 2015-2050, respectively. The subpanels show the factor change in RP for wind speeds equivalent to a category 1 (29 m/s) (**A**) and category 3 (43.4 m/s) (**B**) TC. Gray colors indicate regions with no change.

We also calculate the change in RPs for given wind speed thresholds. The probability of category 1 and category 3 TC wind speeds increases everywhere, except the Bay of Bengal and the Gulf of Mexico (Fig. 4). In the Pacific and North Atlantic basins, the highest positive factor changes for category 3 TCs are generally found toward the boundaries of the regions prone to these wind speeds (between 20°N/S to 40°N/S), amounting to a factor 21 in the Western Pacific. These changes are likely driven by the increase in SSTs in the future-climate GCMs, supporting the poleward extension of intense TCs. However, we also note that TCs in these regions, particularly near 40°N/S, may be prone to extratropical transition, and since this process is not included in STORM, the intensities of TCs in these areas may be poorly represented. Furthermore, we point out that in these regions, the probabilities of a category 3 TC are generally lower, and consequently, a very minor increase in probabilities will result in a large factor change, in contrast to areas that experience such category 3 TCs more frequently. Other TC-prone regions showing a more than 5- to 10-fold increase in the probability of a category 3 strike include Japan, Hong Kong, the Comoros (near Mozambique), and the South Pacific region between 20°S and 30°S. In these areas, the increase in probability is similar across the GCMs (fig. S12). On the other hand, there is a decrease in probability in the Bay of Bengal (Fig. 4); this follows from the shift in genesis locations as discussed above. Similarly, results show little change for the Gulf of Mexico and Caribbean, which is also discussed above.

### Changes in exposed population

Next, we assess changes in exposed population. We find the largest relative increase in population exposed to category 1 RPs below 100 years in Cambodia, with a relative increase of 12,550% compared to the baseline (Table 1). This change is driven by a shift in exposed areas; in the baseline climate, mostly smaller villages along the Cambodian coastline are affected with RPs of category 1 wind speeds below 100 year (total population of around 40,000), whereas under climate change, a much larger area, including the capital city of Phnom Penh (total population exceeding 1 million), is exposed. Note that we deliberately keep the population constant over time, allowing us to solely assess the impact of climate change on exposed populations (see Materials and Methods). Australia faces the largest relative increase in exposed population to category 3 RPs below 500 years, amounting to 9375% (Table 2). Moreover, five of the top 10 countries are located in the South Pacific, the other four countries being Papua New Guinea, New Caledonia, the Solomon Islands, and Tonga. Note that these four countries are all small island developing states, which are typically characterized by high vulnerability to climate impacts, scarce financial resources, and small economies to scale to overcome such impacts (*35*). Eighteen of the 21 countries listed in Tables 1 and 2 that are facing an increase (relative and/or absolute) in exposed population are considered developing countries (*36*).

**Table 1.** Top 10 countries experiencing the largest relative and absolute change in people exposed to category 1 wind speed RPs below 100 years.

	**Country**	**Relative change (%)**	**Country**	**Absolute change (*M*)**
1	Cambodia	12,550	China	153.0
2	Laos	1,514	Vietnam	36.7
3	Mozambique	466	Bangladesh	−23.5
4	Iran	373	United States	18.8
5	Papua New Guinea	237	India	−16.3
6	Palau	116	Somalia	−16.3
7	Vietnam	42	Mexico	7.5
8	Yemen	39	Mozambique	6.5
9	Honduras	−26	Philippines	6.4
10	Myanmar	−26	Cambodia	5.6

**Table 2.** Top 10 countries experiencing the largest relative and absolute change in people exposed to category 3 wind speed RPs below 500 years.

	**Country**	**Relative** **change (%)**	**Country**	**Absolute** **change (*M*)**
1	Australia	9375	China	250.0
2	Yemen	2916	Japan	133.0
3	Papua New Guinea	1442	South Korea	59.4
4	South Korea	935	Bangladesh	−62.4
5	New Caledonia	292	India	−59.3
6	Japan	279	United States	9.9
7	China	161	Mexico	7.1
8	Solomon Islands	135	Madagascar	6.9
9	Venezuela	94	Pakistan	5.0
10	Tonga	92	Myanmar	−5.0

### Coastal regions at risk

**Fig. 5.**
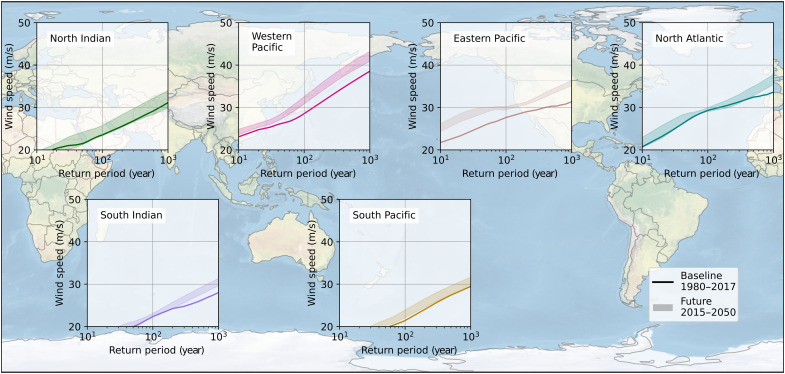
Empirically derived RPs (*x* axis) of 10-min 10-m average wind speeds (*y* axis) weighted by population. The graph represents the average wind speed at a given return RP experienced by an individual living in that basin and exposed to such wind speed probabilities.

To get a basin-wide view of changes in population, we weighted the RP curves by population (combining the information from Fig. 3 and Tables 1 and 2), hereby deriving the average maximum wind speed experienced by a person living near the coast in that basin per RP (Fig. 5). In the Western and Eastern Pacific basins, the future-climate RP curve is visibly higher than the baseline climate curve, indicating that chances of an individual experiencing a stronger TC increase under climate change in all STORM-C datasets. In the other basins, the baseline climate RP curve overlaps with the future-climate range, indicating that climate change will not lead to substantial changes.

## DISCUSSION

We have presented a statistical method for a globally consistent local-scale assessment of changes in future TC risk, both in space and in time. Our methodology combines the benefits of high-resolution GCMs with synthetic TC modeling, overcoming sampling and resolution issues that have limited previous global-scale studies. The resulting open-access synthetic track and wind speed probability maps allow future (2015–2050) local-scale changes in TC frequency and intensity to be more clearly discerned, quantified, and assessed. This allows us to identify hot spot regions facing the largest changes in the probability of being hit by a TC. Our results indicate that the Hong Kong region and the South Pacific are prone to the largest increase in probability for a category 3 TC event. At the same time, there is little change in probabilities in the Gulf of Mexico and a decrease in probabilities for the Bay of Bengal. By combining the RP data with population data, we showed that primarily developing countries, such as Cambodia and Yemen, are prone to large increases in populations exposed to category 1 and category 3 TCs, respectively. To isolate the climate signal, our results were derived by keeping the population data constant. However, population growth and migration toward TC-prone areas are expected to further increase future population exposure (*37*, *38*).

This research represents an important step forward in global TC wind risk assessments by providing new insights into future changes in frequency and intensity of TC events at the local scale. Our open-access datasets can serve as forward-looking hazard maps in catastrophe models used by, for instance, academia, global stakeholders, consultancy, and the (re)insurance industry. Furthermore, this research can contribute to improved quantification of other TC-induced hazards than wind, such as wind-driven waves, storm surge, and precipitation. The procedure presented here is easily applicable to other GCMs, other forcing scenarios, and other time periods and is specifically designed to support consistent comparison across such scenarios and time periods.

## MATERIALS AND METHODS

In this study, we present a statistical method to construct a synthetic TC dataset representative of future-climate conditions. In general, such a dataset can be created in two ways: (i) Extract future-climate TC statistics for each GCM of the multimodel ensemble and run each set through STORM, or (ii) calculate the difference (i.e., delta) between these present- and future-climate TC statistics and add this delta to a baseline dataset (e.g., observed TC statistics). While the first approach is directly applicable to GCM simulation output and therefore does not require the design of additional methodological steps, this approach does not resolve first-order biases of GCMs (such as an underestimation in TC intensity or genesis frequency); rather, it propagates these biases into the synthetic data. Thus, any misrepresentation of TC characteristics in the GCM will be statistically resampled in STORM and can potentially have a substantial effect on the outcomes. On the other hand, the second approach eliminates the effects of the first-order model bias, making it computationally more efficient because the same baseline dataset is used for all GCMs. We do note that in the construction of the delta approach, we assume that the magnitude of such first-order model biases remain unchanged under future warming. It is currently unclear whether and how such biases might alter under climate change; the current best practice is therefore to assume that these biases are constant in time. This implies that we apply the same bias correction to the present- and future-climate GCM datasets. However, when taking the differences between the present- and future-climate GCM datasets, we end up with the same statistics as if we had not bias corrected both datasets. For this reason and under the assumption that the biases do not alter under climate change, we do not apply a bias correction on the GCM datasets. In this section, we first demonstrate why using GCMs in STORM directly results in a poor TC representation; next, we present and validate the so-called delta approach.

### STORM and GCMs

The STORM model takes information on TC track, characteristics, and environmental variables as input variables and statistically resamples these to an equivalent of 10,000 years of TC activity under the same climate conditions. In (*19*), the 1980–2017 (38-year) period of the International Best Track Archive for Climate Stewardship (IBTrACS) (*24*) was used as input to construct a baseline climate synthetic TC dataset (STORM-B) based on observed TC statistics. TC wind speeds were first converted to 10-min 10-m average maximum sustained wind speeds (meters per second), and all data were linearly interpolated to three-hourly values. The STORM model only considers TCs that form within a TC basin domain and in a TC season [see Table 1 in (*19*)]. In the current study, we used these observed TC statistics derived from the IBTrACS dataset (*24*) as the baseline dataset, on which we applied the delta approach.

To determine whether a GCM is suitable for application in our delta approach, we set two conditions. First, the GCM should have both a baseline and a (near-) future model run, of which the baseline (largely) overlaps the 1980–2017 period that was used for the STORM-B dataset. Second, the GCMs need to adequately capture TC activity, so that statistics can be drawn from the data. In this research, we use a high-resolution coupled ocean-atmosphere GCMs that are part of the PRIMAVERA High Resolution Model Intercomparison Project (HighResMIP) multimodel ensemble (*13*, *39*), which, in turn, is part of the Coupled Model Intercomparison Project Phase 6 (CMIP6) (*40*). The GCMs in HighResMIP were solely run using the high-emission SSP585 scenario (*41*, *42*) over the time period 2015–2050; other forcing scenarios and/or time periods are not considered in this HighResMIP protocol. When considering the average climate conditions over 2015–2050 as is done in this study, there is little deviation between the SSP585 scenario and lower-forcing scenarios [the largest deviations start to show after 2050; see also Table SPM.1 in the 2021 Intergovernmental Panel on Climate Change (IPCC) report (*43*)]. We therefore solely use these SSP585 runs for the 2015–2050 period, but we point out that our model setup also allows for other GCMs run with different forcing scenarios and/or time periods.

Of the six coupled ocean-atmosphere GCM runs in HighResMIP, two GCMs are unsuitable for our approach, as these GCMs either lack a future-climate run (ECMWF-IFS) (*44*) or poorly represent TC activity (MPI-ESM1-2) (*45*). We also point out that Roberts *et al*. (*10*) used two different TC tracking algorithms to identify the TCs, namely, TRACK (*46*) and TempestExtremes (*47*). Both algorithms were found to produce similar changes in TC activity under future warming (*10*). Therefore, we do not expect to obtain substantially different results when using both tracking datasets as input here. TRACK, however, was shown to give a better event sampling than TempestExtremes (*10*), giving us more data with which to perform our analysis. We therefore use the publicly available (*48*) TC tracks and characteristics extracted using the TRACK algorithm here. This leaves us with four GCMs, namely, CMCC-CM2-VHR4 (*49*), CNRM-CM6-1-HR (*50*), EC-Earth3P-HR (*51*), and HadGEM3-GC31-HM (*52*). CMCC-CM2-VHR4 has a spatial resolution of 25 km × 25 km in the atmosphere, while the other GCMs have a spatial resolution of 50 km × 50 km. We direct readers to Roberts *et al*. (*10*) for further information on these GCMs. For every GCM, we use the TC data from the periods 1979–2014 (henceforth “present climate”) and 2015–2050 (henceforth “future climate”). These time periods were chosen to (i) ensure a maximum overlap with the IBTrACS dataset and (ii) have an equal temporal length of both the present and future periods.

The GCMs have a 6-hour temporal resolution, which we linearly interpolate to three-hourly values. Following Bloemendaal *et al*. (*19*), we only use TCs that form within the TC basins and within the TC season. However, unlike the procedure explained in Bloemendaal *et al*. (*19*), we do not apply a wind speed threshold of 18 m/s to extract TCs of at least tropical storm force intensity (*26*). Instead, we use all TCs that were identified with the TRACK algorithm. We refrain from this wind speed threshold as GCMs generally underestimate TC intensity; applying a threshold will therefore exclude too many TCs. The TRACK algorithm, however, uses a warm core criterion in its detection algorithm. As a warm core is typically found in TCs of (at least) tropical storm intensity, we believe that the collection of TCs that we use for our analysis meets the input criteria that were also applied to IBTrACS in the creation of the STORM-B dataset. From each of the four GCMs, we extract information on TC genesis frequency (average per year), TC track (longitudinal and latitudinal position of the eye), TC intensity (minimum pressure in hectopascal), and environmental information on SSTs and mean sea level pressure.

To motivate our use of the delta approach, we first demonstrate the direct use of the GCMs in STORM. For four GCM-basin combinations, we compare present-climate statistics against historical data from the IBTrACS dataset (table S3 and fig. S2). In all cases, TCs are substantially weaker in the GCMs than in IBTrACS. We then generate 1000 years of synthetic data using STORM for both the present- and future-climate GCM datasets and observe that the poor representation of TC intensity is carried through the STORM model. For all GCM-basin combinations and for both the present- and future-climate GCM input, the average maximum wind speed along the track is below 30 m/s, whereas this value is around 35 m/s for historical TCs. In addition, the synthetic datasets do not show a (profound) increase in TC intensity under climate change. This shows that the poor representation of TC intensity is propagated through STORM and that it is unadvisable to use TC data from GCM simulations directly as inputs for understanding the impacts of climate change.

### Designing and applying the delta approach

**Fig. 6.**
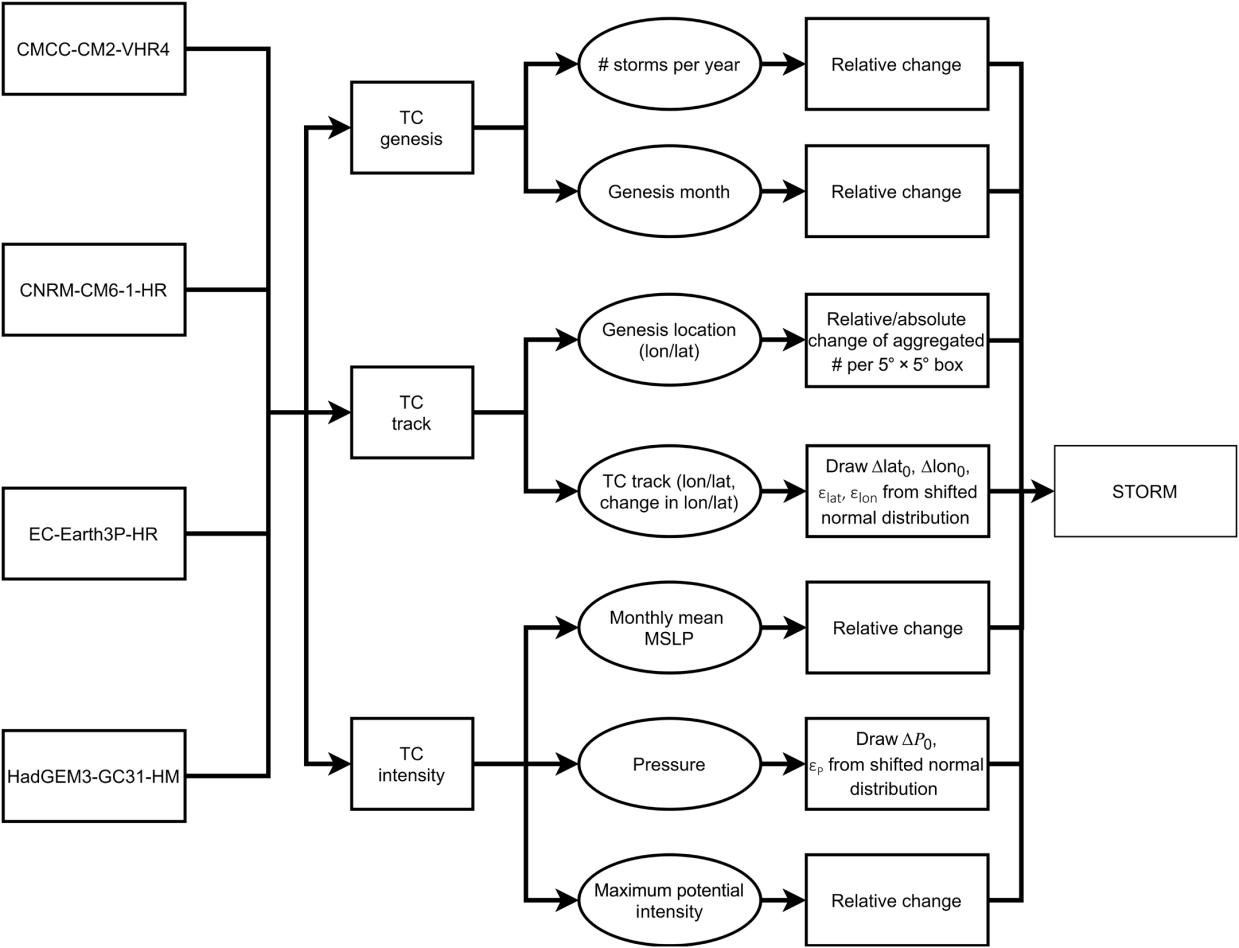
Overview of the propagation of the delta into the STORM model.

We first give an overview of the different (generalized) mathematical equations that form the body of the delta approach. These equations are applied to the following GCM TC statistics (see Fig. 6 for more information): annual frequency, the corresponding genesis months and genesis locations, and changes in track and intensity.

Variables from the present- and future-climate GCM datasets are denoted as ( ∙ )_Present_ and ( ∙ )_Future_,, respectively. The variable from the observational TC dataset (IBTrACS; this dataset served as input for STORM-B) is denoted as ( ∙ )_IBTrACS_, and the variables resulting from adding the delta to the IBTrACS data are denoted as ( ∙ )_IBTrACS, Δ_.

#### Relative changes

Relative changes are calculated and applied to the historical TC statistics from IBTrACS as follows

$${\left(∙\right)}_{\text{IBTrACS},\Delta }={\left(∙\right)}_{\text{IBTrACS}}∙\frac{{\left(∙\right)}_{\text{Future}}-{\left(∙\right)}_{\text{Present}}}{{\left(∙\right)}_{\text{Present}}}+{\left(∙\right)}_{\text{IBTrACS}}$$ (1)

#### Absolute changes

Absolute changes are calculated and applied to the historical TC statistics from IBTrACS as follows

$${\left(∙\right)}_{\text{IBTrACS},\Delta }={\left(∙\right)}_{\text{IBTrACS}}+({\left(∙\right)}_{\text{Future}}-{\left(∙\right)}_{\text{Present}})$$ (2)

#### Shifting the normal distribution

The normal distributions from which variables are drawn are shifted as follows

$$\begin{array}{c} N(\mu_{\text{IBTrACS},\Delta },{\sigma_{\text{IBTrACS},\Delta }}^{2})=\\ N(\mu_{\text{IBTrACS}}+(\mu_{\text{Future}}-\mu_{\text{Present}}),{\sigma_{\text{IBTrACS}}}^{2}∙\frac{{\sigma_{\text{Future}}}^{2}}{{\sigma_{\text{Present}}}^{2}}) \end{array}$$ (3)

where μ denotes the mean, and σ^2^ denotes the variance.

Next, we describe how the delta approach is applied to the different components of STORM (Fig. 6). We highlight the most relevant aspects of the approach. The STORM components and equations discussed below are described in detail in Bloemendaal *et al*. (*19*). Rather than repeating the equations and explanations here, we refer the readers to this companion paper. We also note here that the majority of the approach consists of applying a relative change. This is done because we want to create a future-climate dataset that is relative to the baseline (IBTrACS) dataset.

#### TC genesis

We model the change in genesis frequency λ (average per year) as a relative change, following Eq. 1. To ensure that the number of genesis occurrences aggregated over all months equals the genesis frequency, the shift in genesis frequency per genesis month ψ (average per month) is modeled relative to the total genesis occurrences. Hence, for every month, we calculate the change in genesis frequency relative to IBTrACS (ψ_IBTrACS, Δ_) as follows

$$\begin{array}{cc} \psi_{\text{IBTrACS},\Delta }= & \psi_{\text{IBTrACS}}∙\frac{\#{\text{TCs}}_{\text{Future}(\text{month})}-\#{\text{TCs}}_{\text{Present}(\text{month})}}{\#{\text{TCs}}_{\text{Present}(\text{total})}}\\ & +\psi_{\text{IBTrACS}} \end{array}$$ (4)

#### TC movement

We model the change in genesis locations by first aggregating the number of genesis occurrences in 5° × 5° boxes and then calculating the changes in genesis counts per box. Next, we apply the relative change following Eq. 1. If there are no genesis counts in the present-climate GCM dataset, we calculate and add the absolute change following Eq. 2 instead. In our analysis, these instances occurred mostly poleward of 35°N/S.

Consecutively, the TC track is simulated following the set of regression formulas from James and Mason (*53*) [see Bloemendaal *et al*. (*19*)]. The coefficients in the set of equations are derived directly from the observed TC statistics in IBTrACS. We extract the change in the first-step changes (i.e., the first change after genesis) in longitude and latitude, Δξ_0_ and ∆φ_0_, and the longitudinal and latitudinal residual terms, ε_ξ_ and ε_φ_, from the GCMs. The change in these variables is then applied to the ones derived from IBTrACS using Eq. 3.

#### TC characteristics

TC intensification and weakening are modeled following the set of Eq. 5 in Bloemendaal *et al*. (*19*). The coefficients in these equations are derived directly from the observed TC statistics. The first-step change in pressure, ∆*P*_0_, and the pressure residual term, ε_P_, are extracted from the GCMs. The change in these variables is then applied to the ones derived from IBTrACS following Eq. 3.

The MPI (in hPa) serves as an environmental constraint to the maximum TC intensity at a location and is dependent on the SST at that location. To calculate changes in MPI, we derive the MPI for the GCMs using the SST fields from these GCMs in combination with the coefficients derived from the IBTrACS dataset. Next, we derive the relative changes in MPI per 5° × 5° box and apply this relative change to those in the IBTrACS dataset.

We do not extract information on maximum TC wind speeds from the GCMs because of their biases. Instead, we apply the wind-pressure relationship from Harper (*54*) using the corresponding coefficients that were derived from the observational TC statistics. We do not consider any changes in the radius to maximum winds (*R*_max_), because it is not possible to derive an accurate *R*_max_ value from the GCMs due to the *R*_max_ values being constrained by the 25-km grid resolution of the GCM.

Our delta approach implicitly captures future TC behavior that has been a topic of discussion in recent literature, such as a poleward shift of TC tracks (*31*, *32*, *55*, *56*) or a change in TC translational speed (*57*). A potential poleward extension of TC tracks may follow from the shift in genesis locations in combination with a shift in the first-step changes in longitude and latitude, and the longitudinal and latitudinal residual terms. These latter terms add small perturbations to the track, implying that they can govern a slowdown of the translational speed, an altering in the heading of the TC, or a more poleward movement compared to the baseline climate conditions. Second, the MPI (as was previously discussed) provides an indication of regions supportive of TC development. With increasing SSTs under climate change (*43*), we observe a poleward shift of such supportive regions, resulting in a similar poleward shift of the TC tracks and locations of maximum intensity in the future-climate datasets. In the STORM-C datasets, this effect is predominantly visible in the Western Pacific and the North Atlantic (see fig. S1), in line with other literature (*31*, *32*).

Last, we perform a perfect model run to validate the delta approach. In a perfect model run, we assume that the model itself is “perfect,” i.e., we do not focus on intrinsic model errors but rather assess the influence of the input dataset on the outcomes. Hence, by setting up this perfect model run, we can test whether the delta approach does not lead to anomalies in the output dataset. For the same four GCM-basin combinations used previously, we generate 1000 years of synthetic tracks in two setups: (i) using the present-climate GCM dataset as baseline and adding the delta, and (ii) directly using the future-climate GCM dataset. When comparing the two approaches, table S4 and fig. S3 show that the mean and SDs across all TC variables considered here are almost identical to one another. The TC intensity, measured through the average maximum wind speed along the track, has a maximum deviation of 0.1 m/s for the four GCM-basin combinations. This result implies that the delta approach does not create any anomalies and can be used to generate synthetic TCs for future-climate conditions. Note that the delta approach does not overcome intrinsic model biases in the input dataset: It follows from the intensity statistics in table S4 and fig. S3 that the low TC intensity present in the GCMs is still propagated through STORM.

To quantify the individual influence of the input variables (that are part of the delta approach) on TC intensity, we conduct a sensitivity analysis for one GCM (HadGEM3-GC31-HM) in the Western Pacific. Table S5 and fig. S4 show that the largest influence is seen for the MPI. As this variable serves as an upper bound of TC intensity, it strongly influences the maximum intensity a TC can reach in its lifetime. Figure S4 shows that keeping the MPI constant in a future-climate STORM run strongly decreases the frequency of the most intense (category 5) TCs, reducing them to approximately 1% of all TCs compared to approximately 10% in the STORM-C dataset.

### Calculating RPs

To calculate the wind speed RPs for each of the STORM-C datasets, we follow the same approach as in Bloemendaal *et al*. (*23*). To calculate RPs at a 10-km resolution, we apply the 2D parametric wind field model of Holland (*25*), which has been further refined by Lin and Chavas (*58*), to the future-climate synthetic TC tracks. At each cell of the 10-km grid, we store the maximum wind speed of each TC whenever this wind speed exceeds 18 m/s. Next, the RPs up to 1000 years are calculated empirically using the Weibull plotting formula (*23*, *59*)

$$P_{\text{exc}}(\vec{v})=\frac{i}{n+1}∙\frac{n}{m}$$ (5a)

$$T(\vec{v})=1/P_{\text{exc}}(\vec{v})$$ (5b)

In this equation, $P_{\text{exc}}(\vec{v})$ is the exceedance probability for a given wind speed $\vec{v}$, and *i* is the rank of the respective wind speed, where *i* = 1 is attributed to the highest wind speed. *n* is the length of the set of events, and *m* is the total number of years (here, *m* = 10,000). The RP $T(\vec{v})$ is then calculated as the reciprocal of $P_{\text{exc}}(\vec{v})$. It follows from the set of Equations 5a and 5b that if *n* is large (*n* > 100), then $\frac{n}{n+1}\approx 1$, and the value of $P_{\text{exc}}(\vec{v})$ is dominated by the rank *i*. This implies that, for large *n*, changes in $P_{\text{exc}}(\vec{v})$ for a given event are not due to changes in the total frequency of events *n* but are rather driven by changes in the frequency of exceedance of the respective event, which is represented by its rank *i*.

### Exposure analysis

We assess the impacts of climate change on exposed population by combining the RP maps with global population data (*60*). The population dataset has a 0.125° × 0.125° resolution and is issued for the year 2000 (called as the base year) and for five SSP scenarios, given at decadal intervals between 2010 and 2100. To distinguish between different countries, we overlay the population data with country borders from Natural Earth (www.naturalearthdata.com). We include countries that are considered to be prone to TCs; these countries are generally found between 30°N and 30°S (*61*). To study the sole effect of the future change in TC characteristics on exposure, we aggregate the number of people exposed to category 1 RPs below 100 years and category 3 RPs below 500 years per country. We kept the population data constant at the base year to solely assess the effects of a change in TC wind speed hazard.

Last, we study the maximum wind speed weighted by population per basin. This variable represents the average maximum wind speed at a given RP experienced by an individual living in that basin. To ensure that we are only considering individuals who can potentially face such wind speeds, we include all individuals who are exposed to such wind speeds at an RP level below 1000 years. This way, societies living far inland and not prone to TCs are not included, thereby not skewing the results. We again keep the population data constant at the base year so that emerging differences can solely be attributed to changes in TC wind speeds.

## Supplementary Materials

### This PDF file includes:

Tables S1 to S9 Figs. S1 to S16 References
