# Supplementary material for: A globally consistent local-scale assessment of future tropical cyclone risk
Source: Sci Adv. 2022 Apr 27;8(17):eabm8438. doi: 10.1126/sciadv.abm8438 (PMC9045717; doi:10.1126/sciadv.abm8438)
Supplement: Supplementary file 1 — Tables S1 to S9 Figs. S1 to S16 References [file sciadv.abm8438_sm.pdf]

Science Advances
AAAS

Supplementary Materials for

A globally consistent local-scale assessment of future tropical cyclone risk

Nadia Bloemendaal*, Hans de Moel, Andrew B. Martinez, Sanne Muis,
Ivan D. Haigh, Karin van der Wiel, Reindert J. Haarsma, Philip J. Ward, Malcolm J. Roberts,
Job C. M. Dullaart, Jeroen C. J. H. Aerts

*Corresponding author. Email: nadia.bloemendaal@vu.nl

Published 27 April 2022, *Sci. Adv.* **8**, eabm8438 (2022)
DOI: 10.1126/sciadv.abm8438

This PDF file includes:

Tables S1 to S9
Figs. S1 to S16
References

Supplementary Materials

Here, we provide additional information on the validity of STORM and the STORM+climate change (STORM-C) datasets. We first present the STORM-C statistics for the four GCMs and in all basins. Then, we focus on the validation and justification of our method. We show the STORM model outcomes when directly using the tropical cyclone (TC) data from the global climate models (GCMs). Next, we demonstrate the way the delta approach propagates through STORM, by performing a perfect model run. Subsequently, we test the sensitivity of the model outcomes to various changes in variables by performing a sensitivity analysis.

STORM climate change datasets

Table S1 shows the statistics for the TC characteristics (mean and standard deviation) for the STORM baseline dataset (STORM-B, representative of climate conditions in 1979-2017) and each of the four STORM-C datasets (representative of climate conditions in 2015-2050 under RCP8.5 forcing scenario). Table S2 shows the relative change in TC genesis frequency, frequency of intense (Category 4-5) TCs, and maximum TC intensity between the STORM-B and STORM-C datasets. A discussion of the results is given in **Generation of synthetic tropical cyclones under climate change**.

Table S1 Basin-wide mean and standard deviation (between brackets) of tropical cyclone characteristics of 1,000 36-year samples from the STORM-B and STORM-C datasets. STORM baseline resembles the baseline-climate STORM dataset, generated using IBTrACS and corresponding to the 1980-2017 climate conditions. Similarly, the four "Δ=" columns represent the STORM-C dataset for each of the four GCMS. The last column shows the STORM statistics aggregated over all GCM datasets to show the general direction and magnitude of change. For STORM-C, the second number between brackets indicates how many (out of four) GCMs agree on the sign of change.

		STORM baseline	Δ= CMCC	Δ= CNRM	Δ= EC-Earth	Δ= HadGEM	Aggregated over GCMs
Genesis freq. (avg/yr)	EP	14.5 (0.6)	15.6 (0.6)	14.5 (0.7)	14.9 (0.7)	15.2 (0.6)	15.1 (0.8; 3)
	NA	10.9 (0.6)	12.6 (0.6)	9.3 (0.5)	7.5 (0.4)	9.9 (0.6)	9.8 (1.9; 3)
	NI	2.0 (0.2)	1.7 (0.2)	1.6 (0.2)	2.6 (0.3)	1.8 (0.2)	1.9 (0.4; 3)
	SI	12.3 (0.6)	10.5 (0.6)	11.6 (0.6)	11.2 (0.6)	11.4 (0.6)	11.2 (0.7; 4)
	SP	9.4 (0.5)	9.5 (0.5)	8.2 (0.5)	9.5 (0.6)	10.3 (0.6)	9.4 (0.9; 2)
	WP	23.1 (0.8)	21.9 (0.9)	20.2 (0.8)	23.1 (0.8)	23.5 (0.8)	22.2 (1.5; 2)
	Global	72.4 (1.4)	72.0 (1.5)	65.5 (1.4)	68.8 (1.5)	72.2 (1.5)	69.6 (3.1; 4)
Average pressure along track (hPa)	EP	985.6 (0.4)	979.2 (0.5)	978.1 (0.6)	978.1 (0.6)	977.2 (0.6)	978.2 (0.9; 4)
	NA	985.6 (0.6)	982.7 (0.6)	981.6 (0.8)	980.5 (0.9)	981.1 (0.8)	981.5 (1.1; 4)
	NI	984.3 (1.8)	982.8 (1.8)	981.6 (1.7)	979.9 (1.5)	982.0 (1.9)	981.6 2.0; 4)
	SI	979.5 (0.5)	976.7 (0.6)	975.1 (0.6)	974.9 (0.6)	974.9 (0.6)	975.4 (0.9; 4)
	SP	981.9 (0.5)	977.4 (0.7)	977.8 (0.8)	976.2 (0.7)	976.5 (0.7)	977.0 (1.0; 4)
	WP	974.6 (0.6)	968.4 (0.7)	965.4 (0.8)	967.0 (0.6)	964.9 (0.7)	966.4 (1.5; 4)
	Global	981.9 (4.0)	977.8 (4.9)	976.6 (5.6)	976.1 (4.6)	976.1 (5.7)	976.7 (5.3; 4)
	EP	971.5 (0.8)	960.2 (0.9)	958.4 (1.1)	958.1 (1.1)	956.2 (1.0)	958.2 (1.8; 4)

**Minimum pressure (hPa)**	**NA**	972.7 (1.2)	968.4 (1.1)	966.1 (1.4)	964.5 (1.6)	965.1 (1.5)	966.1 (2.0; 4)
	**NI**	971.6 (3.6)	969.0 (3.3)	967.4 (3.3)	964.5 (2.7)	968.0 (3.5)	967.2 (3.6; 4)
	**SI**	966.0 (0.9)	959.8 (1.1)	957.5 (1.1)	957.6 (1.1)	956.8 (1.1)	957.9 (1.6; 4)
	**SP**	969.4 (1.0)	961.1 (1.2)	961.5 (1.5)	958.8 (1.4)	959.3 (1.3)	960.2 (1.8; 4)
	**WP**	956.3 (1.0)	947.7 (1.1)	943.1 (1.2)	945.5 (1.0)	942.1 (1.2)	944.6 (2.5; 4)
	**Global**	967.9 (5.9)	961.0 (7.2)	959.0 (8.2)	958.2 (6.6)	958.2 (8.7)	959.1 (7.8; 4)
**Maximum wind speed (m/s)**	**EP**	37.0 (0.5)	43.6 (0.5)	44.6 (0.6)	44.8 (0.6)	45.8 (0.6)	44.7 (1.0; 4)
	**NA**	34.7 (0.7)	37.2 (0.7)	38.8 (0.8)	39.6 (0.9)	39.5 (0.9)	38.8 (1.3; 4)
	**NI**	33.4 (1.8)	35.8 (1.8)	36.2 (1.7)	37.9 (1.4)	35.9 (1.8)	36.5 (1.9; 4)
	**SI**	33.9 (0.5)	37.8 (0.6)	38.8 (0.6)	38.6 (0.6)	39.2 (0.6)	38.6 (0.8; 4)
	**SP**	33.4 (0.5)	38.2 (0.6)	38.1 (0.7)	39.4 (0.7)	39.3 (0.6)	38.8 (0.9; 4)
	**WP**	37.6 (0.4)	41.2 (0.5)	43.1 (0.5)	42.4 (0.4)	43.6 (0.5)	42.6 (1.0; 4)
	**Global**	35.0 (1.9)	39.0 (2.8)	39.9 (3.1)	40.4 (2.6)	40.4 (3.4)	39.9 (3.0; 4)
**Landfall counts (avg/yr)**	**EP**	1.4 (0.2)	2.2 (0.3)	2.5 (0.3)	2.2 (0.3)	2.6 (0.3)	2.4 (0.2; 4)
	**NA**	6.0 (0.5)	7.1 (0.6)	5.8 (0.6)	4.9 (0.5)	6.0 (0.6)	6.0 (0.3; 2)
	**NI**	1.2 (0.2)	1.1 (0.2)	1.1 (0.2)	1.7 (0.2)	1.1 (0.2)	1.3 (0.1; 3)
	**SI**	2.6 (0.3)	2.1 (0.3)	2.8 (0.3)	3.4 (0.3)	2.8 (0.3)	2.8 (0.2; 3)
	**SP**	2.7 (0.3)	3.1 (0.4)	2.6 (0.3)	3.2 (0.4)	2.9 (0.4)	2.9 (0.2; 3)
	**WP**	18.3 (1.1)	19.4 (1.1)	19.2 (1.0)	20.4 (1.1)	21.3 (1.1)	20.1 (0.5; 4)
	**Global**	32.2 (1.3)	35.1 (1.3)	33.9 (1.3)	35.9 (1.3)	36.8 (1.4)	35.4 (1.7; 4)
**Landfall pressure (hPa)**	**EP**	983.1 (2.7)	980.6 (2.3)	979.9 (2.4)	981.0 (2.4)	980.6 (2.4)	980.5 (2.4; 4)
	**NA**	985.8 (1.0)	984.4 (1.0)	984.6 (1.3)	983.2 (1.2)	983.9 (1.0)	9840 (1.3; 4)
	**NI**	981.6 (2.4)	983.4 (2.1)	982.6 (1.9)	980.5 (1.7)	981.8 (2.0)	982.1 (2.2; 3)
	**SI**	980.1 (1.2)	978.7 (1.4)	978.3 (1.2)	978.0 (1.1)	977.3 (1.3)	978.1 (1.3; 4)
	**SP**	981.4 (1.5)	977.4 (1.7)	977.9 (1.9)	975.7 (1.8)	975.8 (2.0)	976.7 (2.1; 4)
	**WP**	980.9 (0.7)	977.7 (0.7)	975.4 (0.8)	976.9 (0.7)	975.5 (0.7)	976.4 (1.2; 4)
	**Global**	982.1 (2.5)	980.3 (3.1)	979.7 (3.4)	979.2 (3.0)	978.9 (3.2)	979.5 (3.2; 4)

Table S2 Relative changes (%) in tropical cyclone (TC) frequency, frequency of intense (Category 4-5) TCs, and TC intensity between the baseline climate and each of the four STORM-C datasets.

		**Δ=CMCC**	**Δ=CNRM**	**Δ=EC-Earth**	**Δ=HadGEM**
**Genesis frequency**	**Eastern Pacific**	7.59	0.00	2.76	4.83
	**North Atlantic**	15.60	-14.68	-31.19	-9.17

	**North Indian**	-15.00	-20.00	30.00	-10.00
	**South Indian**	-14.63	-5.69	-8.94	-7.32
	**South Pacific**	1.06	-12.77	1.06	9.57
	**Western Pacific**	-5.19	-12.55	0.00	1.73
	**Global**	-0.55	-9.53	-4.97	-0.28
**Frequency of Cat 4-5**	**Eastern Pacific**	128.66	136.11	146.21	174.77
	**North Atlantic**	33.13	18.74	0.48	35.44
	**North Indian**	3.06	12.12	99.77	24.13
	**South Indian**	59.60	116.20	100.51	131.18
	**South Pacific**	134.06	96.66	191.06	218.18
	**Western Pacific**	37.20	52.95	61.57	82.63
	**Global**	62.94	71.70	80.67	104.64
**TC intensity (maximum wind speed)**	**Eastern Pacific**	17.84	20.54	21.08	23.78
	**North Atlantic**	7.20	11.82	14.12	13.83
	**North Indian**	7.19	8.38	13.47	7.49
	**South Indian**	11.50	14.45	13.86	15.63
	**South Pacific**	14.37	14.07	17.96	17.66
	**Western Pacific**	9.57	14.63	12.77	15.96
	**Global**	11.43	14.00	15.43	15.43

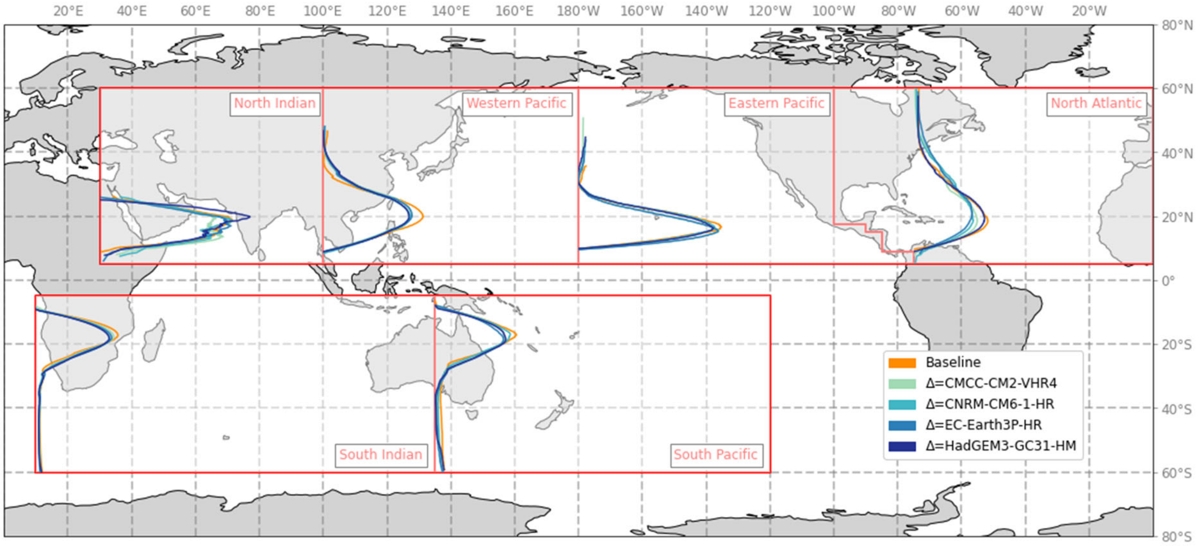

Fig. S1 Latitudinal distribution per basin of the location of maximum intensity in the STORM-B and STORM-C datasets

STORM and global climate model simulations

One of the novel aspects in this study is the methodology of projecting a climate change signal (delta) onto the historical TC data from IBTrACS. The main reason of using IBTrACS is because TCs, and more particularly TC intensity, is poorly captured by GCMs(*39*). To demonstrate this, we first compare the present-climate (1979-2014) statistics of four GCM/basin combinations with statistics from historical data from IBTrACS (1980-2015) ("present climate" column in Table S3). The latter time period was chosen to have an equal temporal length to, and maximum overlap with, the present-climate GCM data. Secondly, we use the present- and future-climate TC statistics from the same GCM-basin

combinations and amplify this to 1,000 years of synthetic data using STORM ("STORM+GCM" column in Table S3) to assess their statistics.

Table S3 Comparison of tropical cyclone characteristics in the global climate models (GCMs). The "baseline climate" column shows the comparison of these characteristics between 36 years of baseline-climate GCM data (1979-2014) and historical data from IBTrACS (1980-2015). The "STORM+GCM" column shows the statistics extracted from 1,000 years of synthetic data, using the baseline (1979-2014) and future (2015-2050) GCM data as input for STORM.

	Basin	GCM	Present climate		STORM + GCM	
			IBTrACS	GCM	Present	Future
Genesis frequency (avg/yr)	Eastern Pacific	CMCC	14.4 (4.1)	30.3 (4.8)	31.7 (5.7)	33.5 (5.3)
	South Indian	CNRM	12.6 (3.9)	18.1 (3.2)	19.1 (4.1)	18.0 (4.2)
	North Atlantic	EC-Earth	10.6 (4.0)	5.9 (2.5)	7.2 (2.6)	5.2 (2.0)
	Western Pacific	HadGEM	22.2 (4.0)	35.8 (5.5)	37.4 (6.2)	38.4 (6.1)
Average pressure (hPa)	Eastern Pacific	CMCC	988.6 (11.8)	1003.5 (6.4)	994.9 (5.2)	994.8 (4.6)
	South Indian	CNRM	980.2 (12.1)	1003.3 (7.0)	985.4 (7.0)	986.0 (6.5)
	North Atlantic	EC-Earth	991.6 (12.7)	1007.7 (4.1)	994.3 (3.2)	994.2 (3.8)
	Western Pacific	HadGEM	978.7 (14.8)	998.8 (6.2)	984.0 (11.4)	983.4 (14.4)
Minimum pressure (hPa)	Eastern Pacific	CMCC	973.9 (25.0)	989.6 (17.1)	989.2 (11.6)	989.3 (8.8)
	South Indian	CNRM	961.9 (25.4)	990.4 (16.4)	978.8 (12.9)	979.6 (12.4)
	North Atlantic	EC-Earth	977.3 (25.1)	998.3 (10.6)	992.2 (5.5)	992.1 (6.9)
	Western Pacific	HadGEM	961.5 (28.2)	985.9 (13.7)	975.2 (20.3)	973.4 (25.9)
Maximum wind speed (m/s)	Eastern Pacific	CMCC	35.9 (13.5)	24.9 (6.9)	26.2 (7.2)	26.5 (6.4)
	South Indian	CNRM	36.0 (12.7)	23.6 (7.7)	26.4 (7.6)	25.8 (7.1)
	North Atlantic	EC-Earth	34.6 (13.1)	18.3 (4.5)	20.7 (3.8)	20.8 (4.9)
	Western Pacific	HadGEM	35.7 (11.9)	23.8 (5.5)	28.5 (9.4)	29.3 (11.1)
Landfall counts (avg/yr)	Eastern Pacific	CMCC	1.9 (1.7)	9.2 (4.5)	2.8 (1.9)	3.8 (2.3)
	South Indian	CNRM	4.4 (2.5)	0.0 (0.0)	7.1 (3.2)	6.3 (2.8)
	North Atlantic	EC-Earth	7.8 (5.1)	11.1 (5.9)	2.0 (1.6)	1.4 (1.3)
	Western Pacific	HadGEM	18.7 (4.5)	27.5 (22.1)	28.3 (8.6)	26.0 (8.2)
Landfall pressure (hPa)	Eastern Pacific	CMCC	982.6 (14.6)	1003.5 (12.9)	989.7 (16.9)	993.7 (10.4)
	South Indian	CNRM	976.1 (19.9)	-	985.3 (7.1)	985.8 (6.9)
	North Atlantic	EC-Earth	984.0 (19.4)	1008.7 (4.5)	994.0 (4.8)	994.0 (3.9)
	Western Pacific	HadGEM	978.9 (15.9)	996.5 (11.0)	983.4 (11.6)	983.2 (13.6)

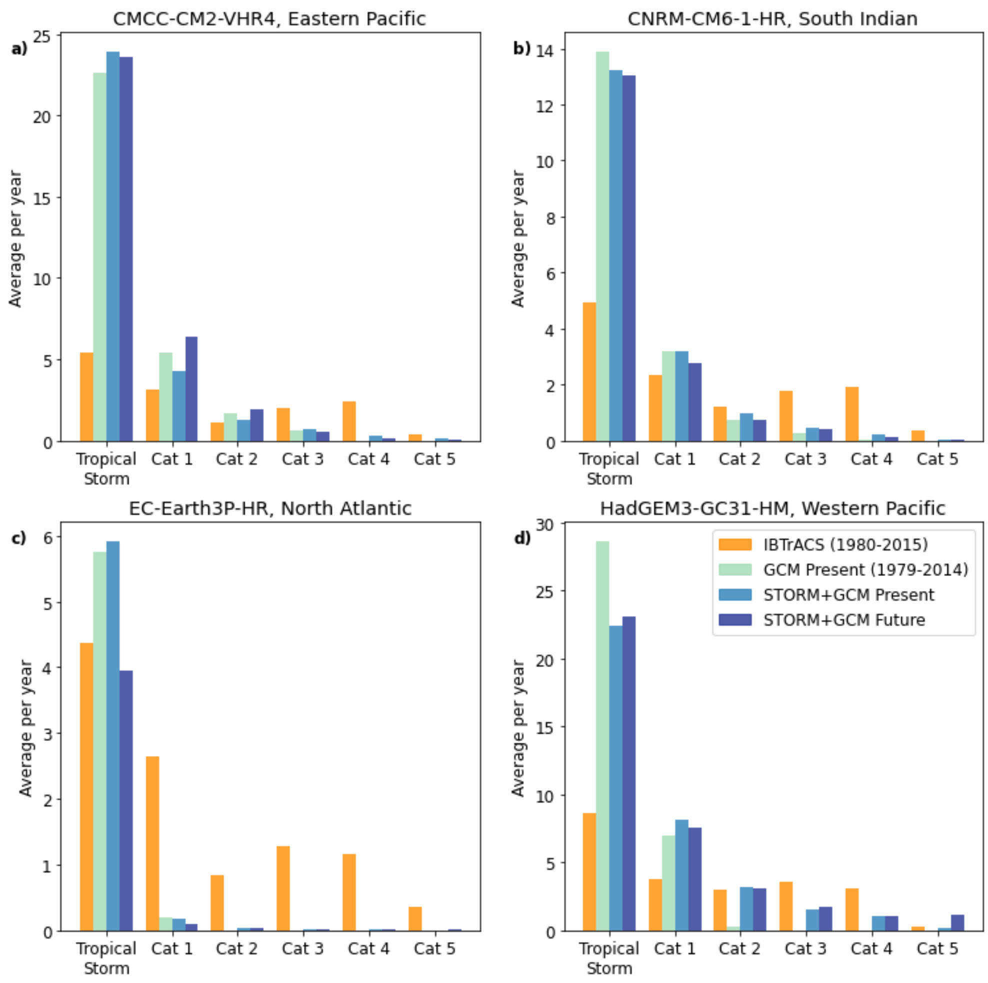

Fig. S2 Distribution of tropical cyclone categories (on the Saffir-Simpson scale) in each of the four model/basin combinations. Shown are the present-climate dataset comparisons (historical data from IBTrACS versus climate data from the global climate models –GCMs-), and 1,000 years of synthetic data using the present- and future-climate GCM runs as input for STORM.

For the present-climate comparison, we observe that there is little consensus in the specific basins between the GCMs and the historical data: in all cases, TCs are substantially weaker in the GCMs as compared to IBTrACS. Furthermore, there is also little agreement on the genesis frequency: in three out of four GCM/basin combinations, there is an overestimation of TC occurrences compared to historical data. The largest difference can be seen for the CMCC model, where there are on average twice as much TC formations in the Eastern Pacific than in observations. Contrarily, there are almost twice as little formations in the North Atlantic in the EC-Earth model. In the synthetic datasets (STORM+GCM column) we observe that the weak TC intensities are preserved by STORM, as for instance the average maximum wind speed is well below hurricane strength (<29 m/s), whereas the average TC in IBTrACS reaches wind speeds of around 35 m/s (Category 1 on the Saffir-Simpson scale). By design, STORM statistically resamples the statistics found in original input datasets, and as such the poor representation of TC intensity in the GCMs is propagated through STORM. Hence, using these GCMs as input for STORM will result in a (severe) underestimation of TC intensity, in turn affecting TC wind speed probabilities and risk assessments, especially for stronger storms.

Perfect model run

To overcome the limitations imposed by the GCMs, we need to diverge from directly using the GCM data, and instead create a STORM input dataset based on the observed TC statistics from IBTRACS. This way, we can ensure that we have a realistic number of TC formations, and that the statistics of

stronger TCs are also included in STORM. Therefore, in this study, we adjust IBTrACS such to include the delta derived from the four GCMs, this way creating a “future climate” version of the historical statistics. To test if introducing such delta does not instigate anomalies, we perform a perfect model run using a combination of the four GCMs and four basins as input. In a perfect model run, we assume that the model itself is “perfect”, that is, we do not focus on intrinsic model errors, but rather assess the influence of the input dataset on the outcomes.

Table S4 Average of 36.000 years, sampled from 1,000 years of synthetic data from each of the models in the indicated basin. In run 1, we extract the present-climate variables and add the climate signal (Δ) onto them to produce a “future-climate version” of this present-climate dataset. In run 2, we directly use the future-climate variables. In both runs, we use the regression formula coefficients from the present-climate datasets.

	Basin	Model	Run 1: Present climate + Δ	Run 2: Future climate
**Genesis frequency (avg/yr)**	**Eastern Pacific**	CMCC	33.3 (5.6)	33.7 (5.6)
	**South Indian**	CNRM	18.0 (4.3)	18.0 (4.2)
	**North Atlantic**	EC-Earth	5.1 (1.9)	5.2 (2.1)
	**Western Pacific**	HadGEM	38.3 (6.2)	38.1 (6.1)
**Average pressure (hPa)**	**Eastern Pacific**	CMCC	994.2 (5.6)	994.2 (5.6)
	**South Indian**	CNRM	985.5 (6.7)	985.4 (6.8)
	**North Atlantic**	EC-Earth	994.0 (3.6)	993.9 (4.0)
	**Western Pacific**	HadGEM	985.4 (9.9)	985.3 (10.0)
**Minimum pressure (hPa)**	**Eastern Pacific**	CMCC	987.8 (12.2)	987.7 (12.5)
	**South Indian**	CNRM	978.7 (12.5)	978.7 (12.6)
	**North Atlantic**	EC-Earth	991.6 (6.1)	991.3 (7.1)
	**Western Pacific**	HadGEM	977.2 (18.0)	977.2 (18.2)
**Maximum wind speed (m/s)**	**Eastern Pacific**	CMCC	27.5 (8.0)	27.5 (8.1)
	**South Indian**	CNRM	26.6 (7.5)	26.6 (7.5)
	**North Atlantic**	EC-Earth	21.3 (4.6)	21.4 (5.2)
	**Western Pacific**	HadGEM	27.9 (8.7)	27.8 (8.7)
**Landfall counts (avg/yr)**	**Eastern Pacific**	CMCC	4.8 (2.6)	4.6 (2.4)
	**South Indian**	CNRM	7.1 (3.2)	7.3 (3.1)
	**North Atlantic**	EC-Earth	1.7 (1.6)	1.6 (1.5)
	**Western Pacific**	HadGEM	27.4 (8.7)	27.1 (8.5)
**Landfall pressure (hPa)**	**Eastern Pacific**	CMCC	991.3 (15.0)	990.7 (15.9)
	**South Indian**	CNRM	985.4 (7.0)	985.4 (6.8)
	**North Atlantic**	EC-Earth	993.4 (4.0)	993.4 (4.1)
	**Western Pacific**	HadGEM	983.6 (11.2)	983.6 (11.4)

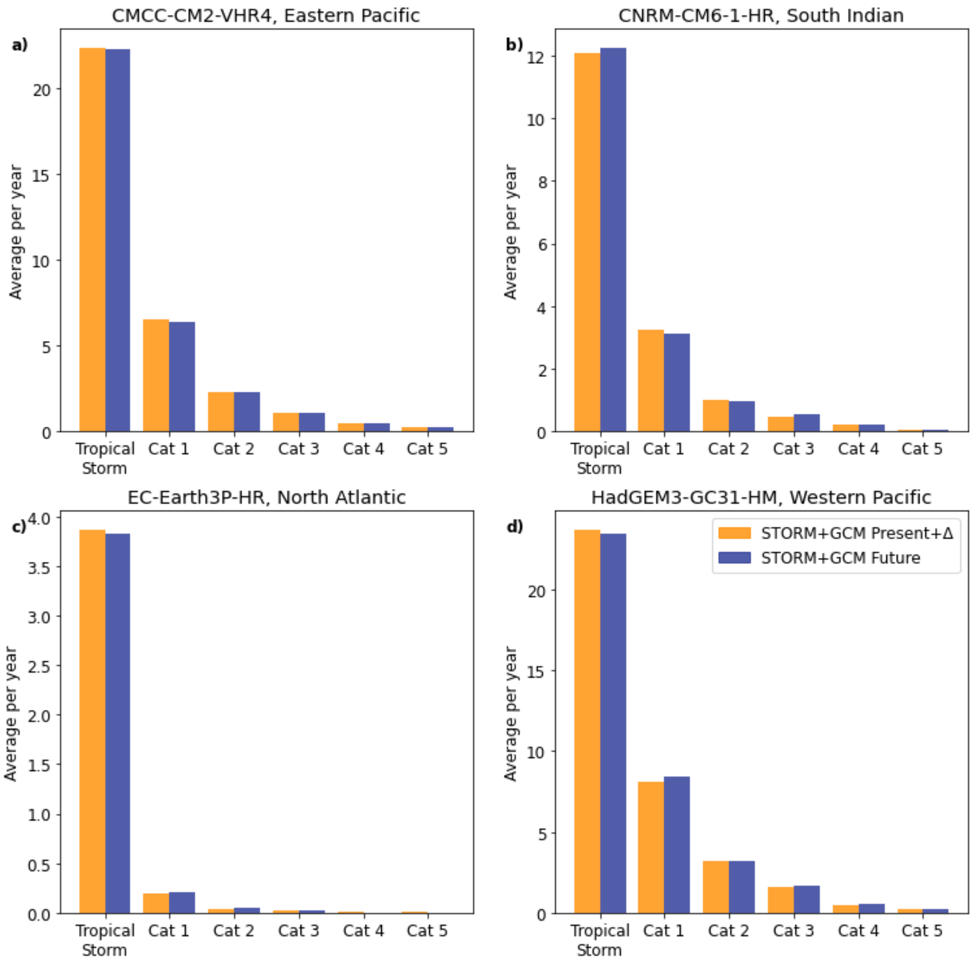

Fig. S3 Distribution of tropical cyclone categories (average per year) on the Saffir-Simpson scale in 1,000 random realizations of 36 years of data drawn from 1,000 years of synthetic data from STORM for the present (orange) and future climate (turquoise). Results are shown for (a) CMCC-CM2-VHR4 in the Eastern Pacific, (b) CNRM-CM6-1-HR in the South Indian, (c) EC-Earth3P-HR in the North Atlantic, and (d) HadGEM3-GC31-HM in the Western Pacific**.**

In the data preprocessing step in STORM, we extract the various variables and variable distributions, as well as determine the coefficients for the regression formulas; see Fig. 6 for an overview of all variables, and Bloemendaal et al. (*19*) for a full description of all variables and coefficients. Additionally, we also extract the delta from the four GCMs following the approach set out in **Materials and Methods**. Next, to test whether the delta approach is stable and does not introduce anomalies, we perform two runs in which we use the coefficients from the present-climate GCM dataset, but vary the variables as follows: i) in the first run, we use the TC statistics from the present-climate GCM simulation, but add the delta to create a future-climate version of these present-climate statistics (we call this "present+Δ"). This approach mimics the methodology we apply to the observed TC statistics from the IBTrACS dataset; and ii) in the second run, we directly use the TC statistics from the future-climate GCM simulation. If the present +Δ run performs well, the outcomes of the first run should compare well to the second one. These two run setups serve as input for STORM, and are used to generate 1,000 of synthetic data for four GCM/basin combinations. Subsequently, we randomly sample 1,000 times 36 years of data (to comply with the temporal length of the original GCM simulations; this totals to 36,000 years of data), and extract the yearly number of genesis and landfall occurrences, as well as the of various TC intensity measures. We then calculate the mean and standard deviation of each of the six characteristics, see Table S4. We observe that the means and standard deviations of the two runs are almost identical to one another, which implies that adding the delta does not impose

anomalies and thus that the delta approach is stable, mimicking the results that we would obtain if we were to use the TC statistics directly from a future-climate GCM dataset as input (run 2).

Fig. S3 shows the distribution of TC categories for each of the four GCM/basin combinations for the present+Δ and future-climate STORM simulations in the perfect model setup. As was the case with the statistics of the various TC characteristics (see Table S1) we also see that the average annual number of TCs per category is approximately equal in each of the category bins, meaning that the present+Δ perfect model run again mimics the results if we had used the future-climate GCM TC statistics instead, and that no anomalies are brought into the synthetic STORM datasets.

Sensitivity analysis

To analyze the influence of applying the delta approach on the different variables (see Fig. 6 in **Materials and Methods**) on the overall outcome of the STORM runs, we perform a sensitivity analysis using the TC statistics from HadGEM in the Western Pacific. The reason we use this GCM in this basin is because it consists of the most TC data of all GCM/basin combinations, and the Western Pacific generally faces some of the strongest TCs of all basins.

Table S5 Statistics of six key tropical cyclone characteristics for the baseline STORM run (STORM-B), the STORM-climate change run (STORM-C; IBTrACS+Δ), and the ten synthetic runs in which the named variable was kept constant (i.e. the baseline-climate value) and not shifted according to the delta approach. Average values of 1,000 times 36 years of data (36,000 years) are given, standard deviations are listed between brackets

	Genesis frequency (avg/yr)	Average pressure (hPa)	Minimum pressure (hPa)	Maximum wind speed (m/s)	Landfall counts (avg/yr)	Landfall pressure (hPa)
**STORM-B**	23.9 (4.9)	974.6 (16.1)	956.4 (28.5)	37.5 (12.1)	18.1 (6.1)	980.9 (13.6)
**STORM-C**	24.5 (5.0)	965.0 (19.8)	942.4 (31.9)	43.5 (12.7)	21.4 (6.8)	975.5 (15.0)
$\Delta lat_0, \Delta lon_0$	24.4 (5.0)	965.2 (19.8)	942.7 (32.0)	43.4 (12.8)	21.3 (6.5)	975.6 (15.2)
ΔP_0	24.3 (4.9)	965.0 (19.8)	942.3 (32.0)	43.6 (12.8)	21.4 (6.6)	975.3 (15.2)
ε_P	24.2 (4.9)	964.8 (19.9)	942.1 (32.0)	43.6 (12.8)	21.4 (6.5)	975.4 (15.3)
$\varepsilon_{lat}, \varepsilon_{lon}$	24.0 (4.9)	964.7 (20.0)	942.0 (31.9)	43.7 (12.7)	21.1 (6.5)	975.5 (15.3)
**Genesis frequency**	23.7 (4.9)	964.9 (19.8)	942.1 (32.0)	43.6 (12.8)	20.7 (6.7)	975.1 (15.4)
**Genesis location**	24.0 (5.0)	966.2 (19.7)	944.4 (31.7)	42.7 (12.8)	21.3 (6.7)	976.3 (14.8)
**Genesis month**	24.5 (4.7)	965.1 (19.8)	942.3 (32.0)	43.6 (12.7)	21.5 (6.8)	975.5 (15.1)
**Maximum Potential Intensity**	24.6 (4.9)	969.4 (16.5)	948.7 (27.7)	41.1 (11.4)	19.6 (6.4)	979.1 (13.3)
**Environmental pressure**	24.3 (4.8)	964.8 (19.8)	942.2 (32.0)	43.5 (12.8)	21.1 (6.8)	975.4 (15.3)
$\Delta P_{.01}, \Delta P_{.99}$	24.3 (5.1)	964.9 (19.8)	942.2 (32.0)	43.6 (12.8)	21.6 (6.7)	975.5 (15.0)

The statistics (mean and standard deviation) of six tropical cyclone characteristics for each of the sensitivity test runs are listed in Table S5. Fig. S4 shows the distribution of TC categories in the STORM-B dataset, the STORM-C dataset, and the ten synthetic runs in which we kept each of the ten input variables constant (that is, we used the baseline-climate value and did not shift it using the delta approach set out in **Materials and Methods**) while shifting the other variables. As such, we can analyze the individual influence of each of the variables on the STORM-C dataset. From Fig. S4, we see that most of the variables on itself do not influence the mean value of each of the characteristics, but the only variable that does is the Maximum Potential Intensity (MPI, in hPa). This variable is a measure of the theoretical maximum TC intensity possible at a location, dependent on environmental factors such as sea-surface temperature (SST) and the environmental pressure. It directly influences TC intensification and weakening, as TCs will weaken as soon as they approach the MPI, and when they reach higher latitudes, where SSTs are lower (*67*). From Table S5 we can observe the influence of the

MPI on the maximum intensity of a TC; the baseline climate MPI leads to approximately 2.4 m/s lower maximum wind speeds compared to its future-climate counterpart in the STORM-C dataset. This has a direct influence on the distribution of TC categories (Fig. S4), where we observe a substantial drop in Category-5 occurrences, similar to the STORM-B dataset. This means that the MPI predominantly affects the occurrence of the strongest TCs.

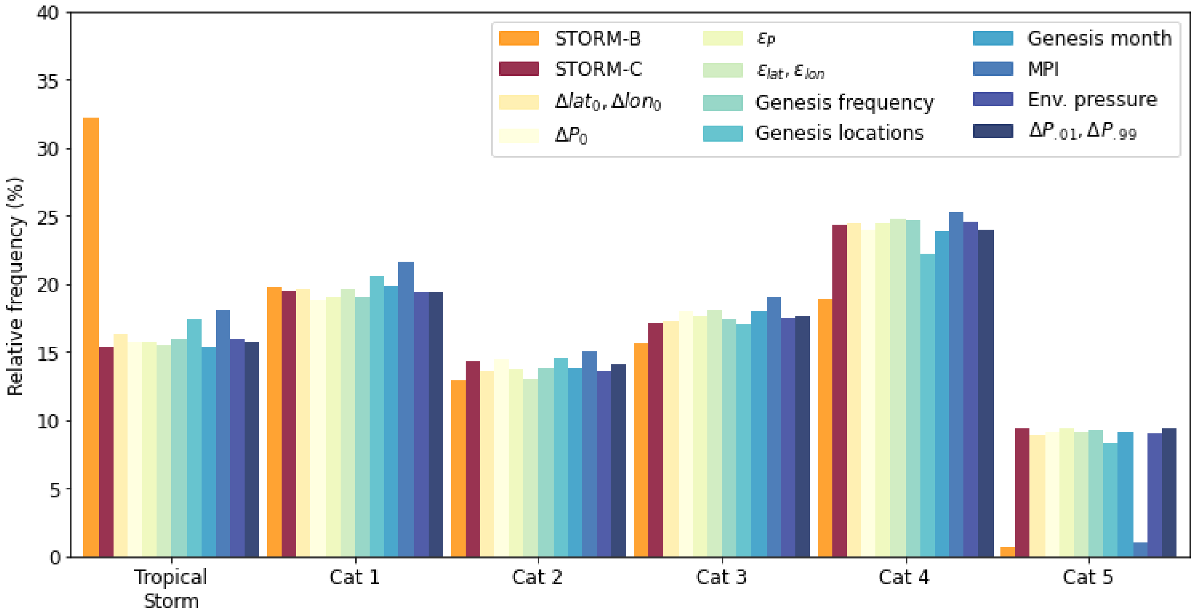

Fig. S4 Relative frequency of tropical cyclone categories for the STORM baseline climate run (STORM-B), the STORM-climate change run (STORM-C), and the ten synthetic runs in which the named variable was kept constant (i.e. the baseline climate value) and not shifted according to the delta approach.

Uncertainty analysis

To provide insights in the spread of two key variables in the delta approach, we perform a bootstrap procedure on the Poisson parameter (TC frequency) and the Maximum Potential Intensity (a measure of TC intensity).

Table S6 Uncertainty estimates for tropical cyclone frequency, here given as the Poisson parameter that goes into STORM after applying the delta approach on the Poisson parameter value in IBTrACS. Values listed represent the mean Poisson parameter (with equivalent definition) over 1,000 iterations with replacement; standard deviation is listed between brackets.

	Eastern Pacific	North Atlantic	North Indian	South Indian	South Pacific	Western Pacific
**CMCC**	15.5 (0.5)	12.5 (1.7)	1.7 (0.2)	10.5 (0.6)	9.4 (0.5)	21.5 (1.0)
**CNRM**	14.5 (0.7)	9.1 (0.5)	1.6 (0.1)	11.6 (0.5)	8.1 (0.4)	19.9 (1.0)
**EC-Earth**	14.8 (0.7)	7.3 (0.8)	2.6 (0.3)	11.0 (0.6)	9.5 (0.6)	22.7 (1.3)
**HadGEM**	15.1 (0.5)	9.7 (0.5)	1.8 (0.2)	11.3 (0.5)	10.2 (0.3)	23.0 (0.8)

Table S7 Uncertainty estimates for the basin-wide relative change in Maximum Potential Intensity in the basin's most intense month. Values listed represent the mean relative change between the GCM's present- and future-climate dataset over 1,000 iterations with replacement; standard deviation is listed between brackets. Please note that we provide this basin-wide relative change to condense a larger set of spatially varying changes (which are actually the ones used in the delta approach) into one number; actual relative changes at the local scale within a basin can deviate from the numbers listed here.

	Eastern Pacific	North Atlantic	North Indian	South Indian	South Pacific	Western Pacific
**CMCC**	-0.62 (0.09)	-0.90 (0.08)	-1.22 (0.26)	-0.40 (0.05)	-0.45 (0.07)	-0.77 (0.09)
**CNRM**	-1.39 (0.12)	-0.87 (0.09)	-2.48 (0.24)	-0.53 (0.05)	-0.75 (0.08)	-1.36 (0.12)
**EC-Earth**	-0.90 (0.1)	-0.97 (0.08)	-2.30 (0.32)	-0.59 (0.07)	-0.81 (0.1)	-0.95 (0.09)
**HadGEM**	-1.57 (0.11)	-1.42 (0.09)	-3.50 (0.29)	-0.71 (0.06)	-1.34 (0.09)	-1.70 (0.12)

Return period analysis – wind speeds for fixed return periods

Figs. S5 and S6 show the maximum wind speeds for the fixed return periods discussed in the main paper for the individual GCMs. Fig. S7 shows the relative change in wind speed at the 100- and 1,000-yr return period level. Fig. S8 shows number of GCMs agreeing on the sign of change for the different return periods – this number provides an indication of the confidence in the sign of change. Fig. S9 shows the largest and smallest differences in maximum wind speed; Fig. S10 portrays to which GCM these largest and smallest changes are attributed to. Tables S8 and S9 provide basin-wide statistics of the change in 100- and 1,000-yr maximum wind speeds as is visualized in Fig. 2.

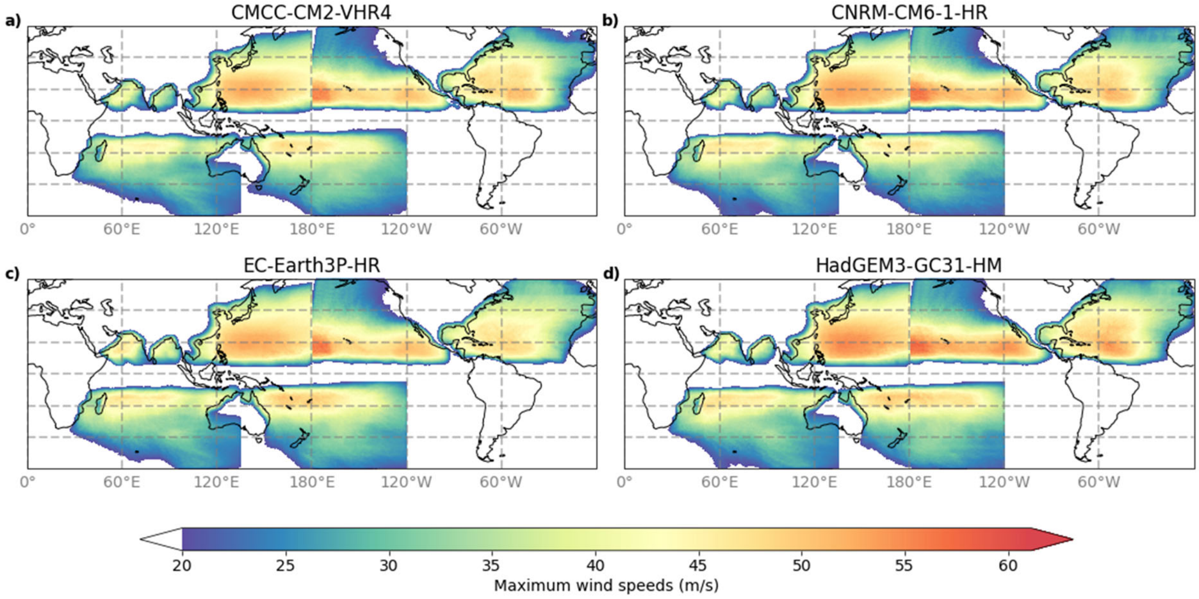

Fig. S5 Spatial distribution of maximum 10-minute 10-meter wind speeds at the 100-yr return period in each of the four global climate models. The wind speeds are the average value of 1,000 random realizations of 10,000 years of data (sampled with replacement)

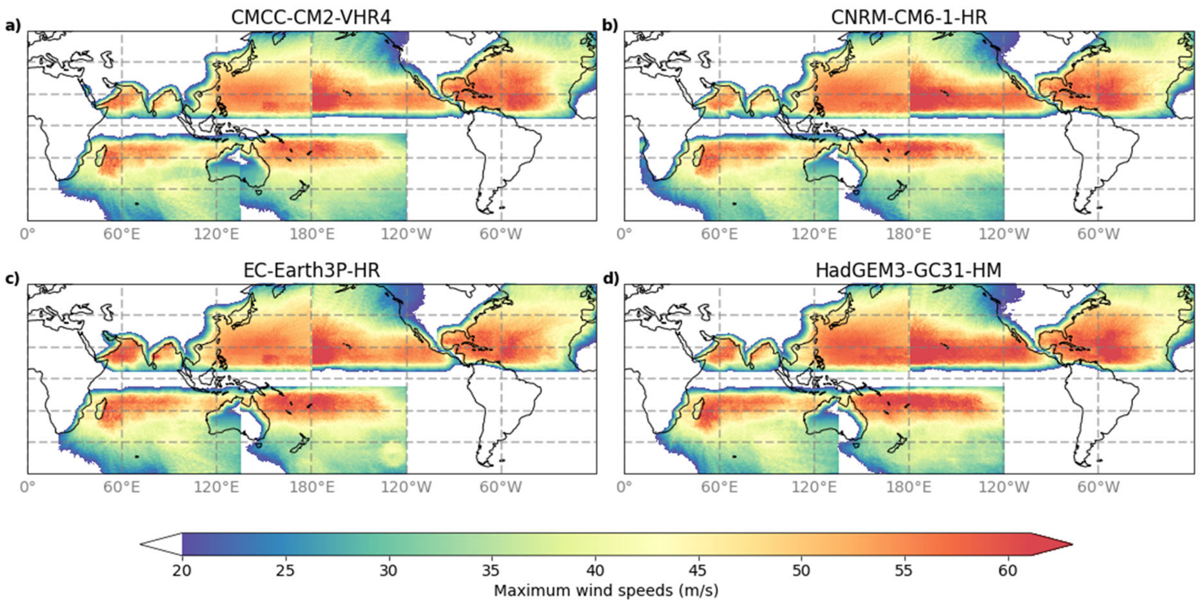

Fig. S6 Same as Fig. S5, but now for the 1,000-yr return period.

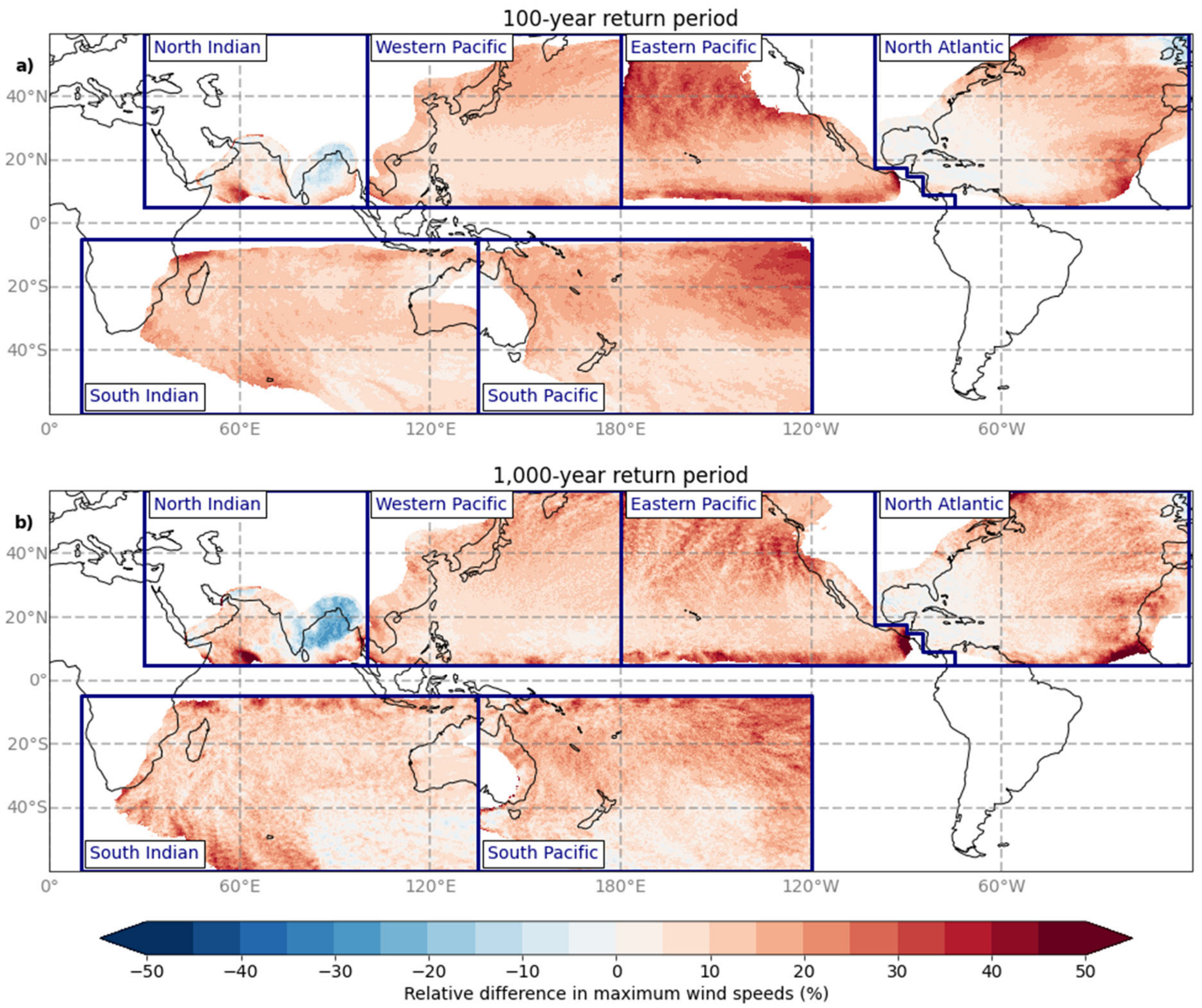

Fig. S7 Relative change in 10-minute 10-meter average maximum wind speed between STORM-B (corresponding to the average climate conditions of 1980-2017) and the ensemble median of the STORM-C datasets (corresponding to the average climate conditions of 2015-2050) for the 100-yr (a) and 1,000-yr (b) return period. Red tones indicate a positive change (i.e. an increase in wind speed), blue tones indicate a negative change.

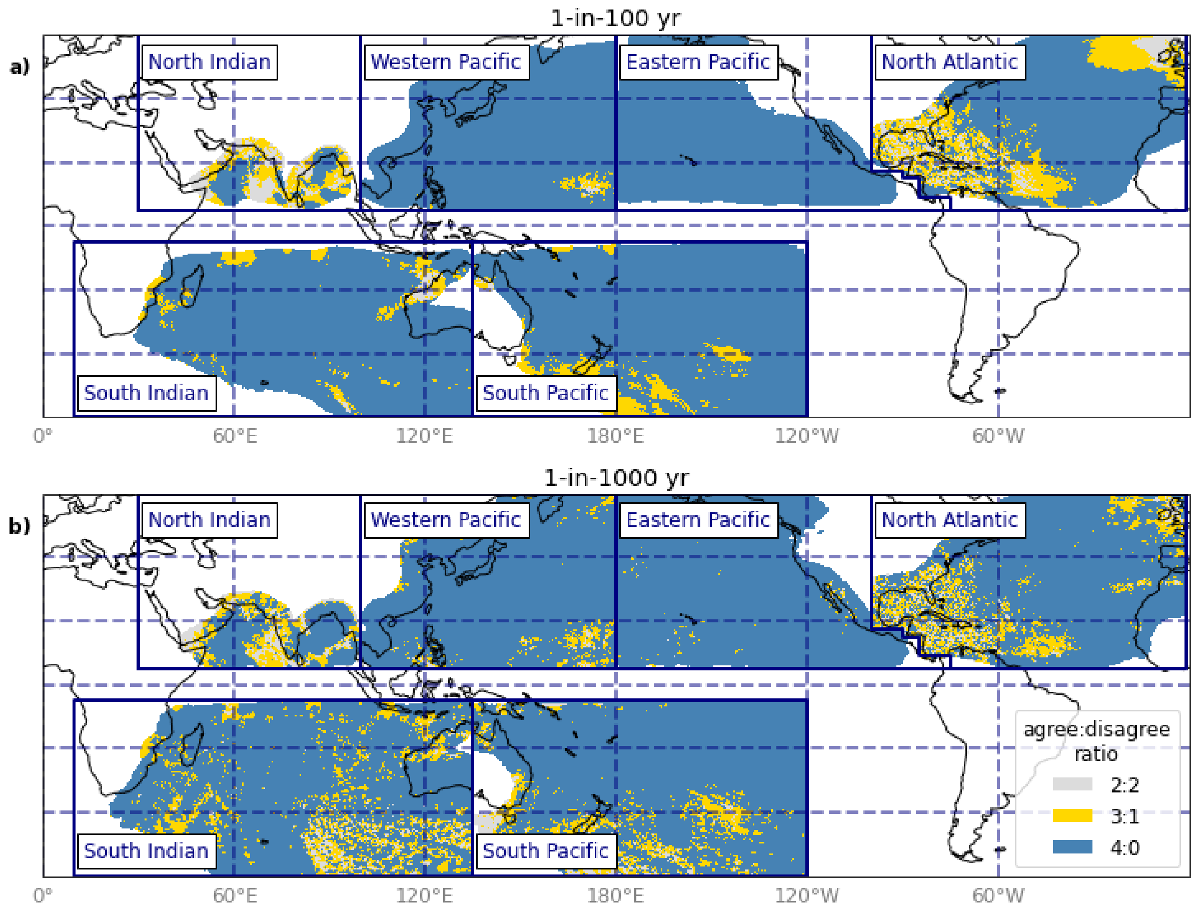

Fig. S8 Number of STORM climate change datasets (one per global climate model; four in total) agreeing on the sign of change compared to the STORM baseline climate datasets.

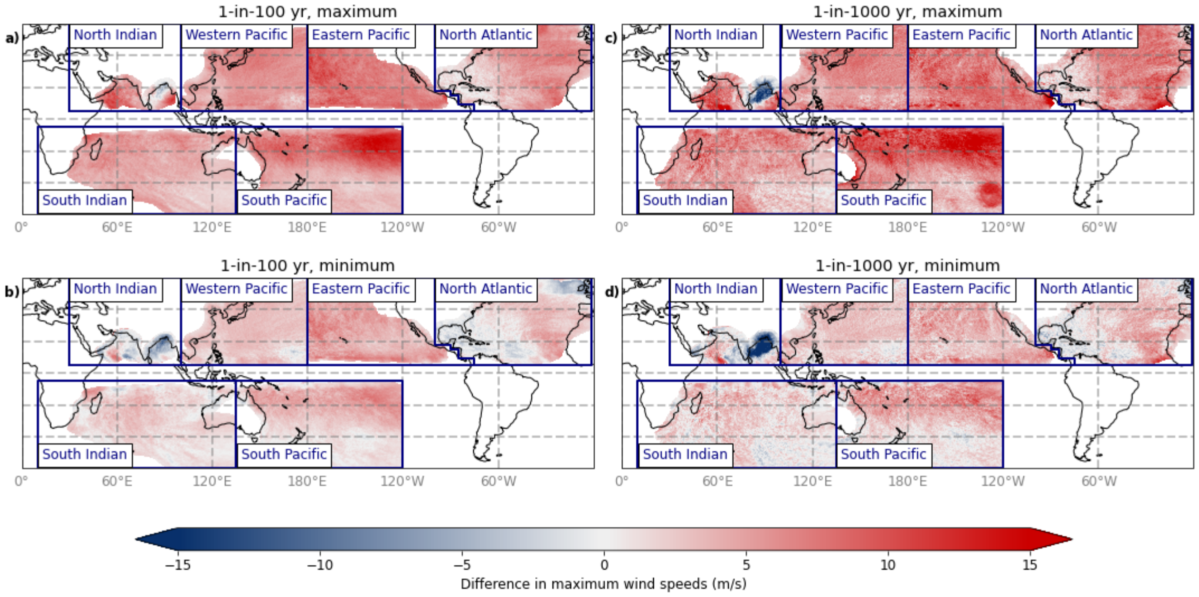

Fig. S9 Maximum (top row) and minimum (bottom row) value of the difference in maximum wind speeds compared to the STORM baseline climate dataset.

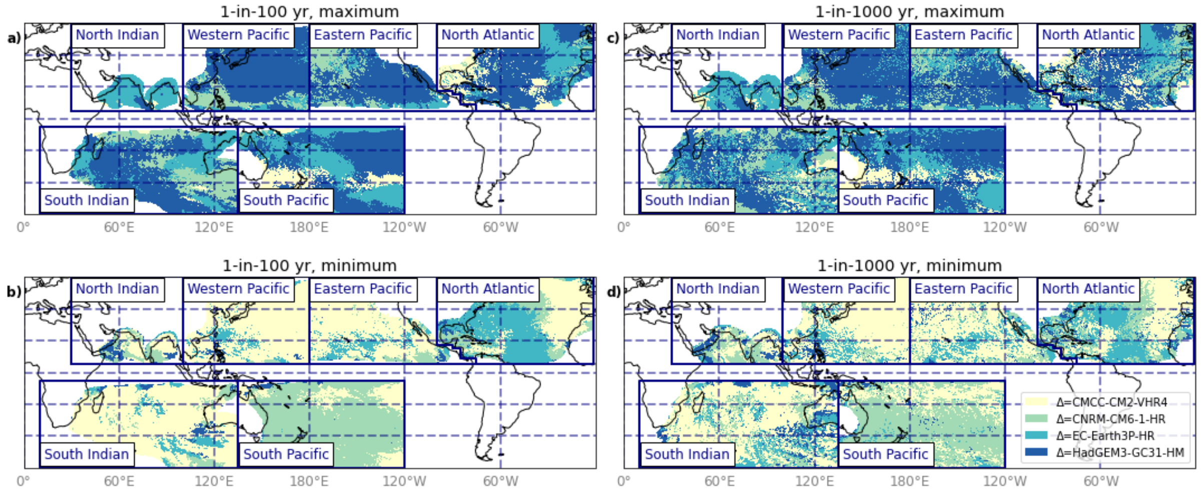

Fig. S10 Global climate model having the maximum (top) and minimum (bottom) value of the difference in maximum wind speed compared to the STORM baseline climate dataset.

Table S8 Basin-wide statistics of the changes in 10-meter 10-minute average maximum wind speeds (in m/s) at the 100-yr return period between STORM-B (corresponding to the average climate conditions of 1980-2017) and the ensemble median of the STORM-C datasets. These statistics are calculated over the data as visualized in Fig. 2a.

	Eastern Pacific	North Atlantic	North Indian	South Indian	South Pacific	Western Pacific
**Median**	5.85	3.57	0.94	3.06	3.81	4.10
**Mean**	5.97	3.09	0.79	2.98	4.23	4.04
**St. dev.**	1.70	2.24	2.65	1.17	2.10	1.01
**25 and 75% percentile**	4.93 - 6.85	1.28 - 4.70	-0.65 - +2.27	2.07 - 3.80	2.36 - 6.12	3.47 - 4.76
**Minimum**	0.00	-5.55	-8.51	-1.08	-1.42	-1.08
**Maximum**	25.76	24.50	10.25	7.81	9.73	6.88

Table S9 Same as Table S8, but now for the 1,000-yr return period (Fig. 2b).

	Eastern Pacific	North Atlantic	North Indian	South Indian	South Pacific	Western Pacific
**Median**	5.44	4.23	1.65	3.29	4.44	4.35
**Mean**	5.53	3.96	0.06	3.23	4.80	4.24
**St. dev.**	2.21	2.74	6.42	1.78	3.23	1.55
**25 and 75% percentile**	4.39 - 6.48	2.13 - 5.67	-2.03 - +4.11	1.94 - 4.45	2.31 - 7.07	3.28 - 5.22
**Minimum**	-1.70	-7.63	-20.94	-3.20	-4.92	-6.52
**Maximum**	35.41	34.0	15.37	13.95	34.50	27.38

Return period analysis – return periods for fixed wind speeds

Figs S10 and S11 show the return periods for the fixed TC categories discussed in the main paper for the individual GCMs. Fig. S12 shows the number of GCMs agreeing on the sign of change for the Category-1 and Category-3 return periods – this number provides an indication of the confidence in the sign of change. Fig. S13 shows the largest and smallest factor change in return periods; Fig. S14 portrays to which GCM these largest and smallest changes are attributed to. Tables S8 and S9 provide basin-wide statistics of the factor change in return periods for a Category-1 and Category-3 event, respectively, as is visualized in Fig. 4.

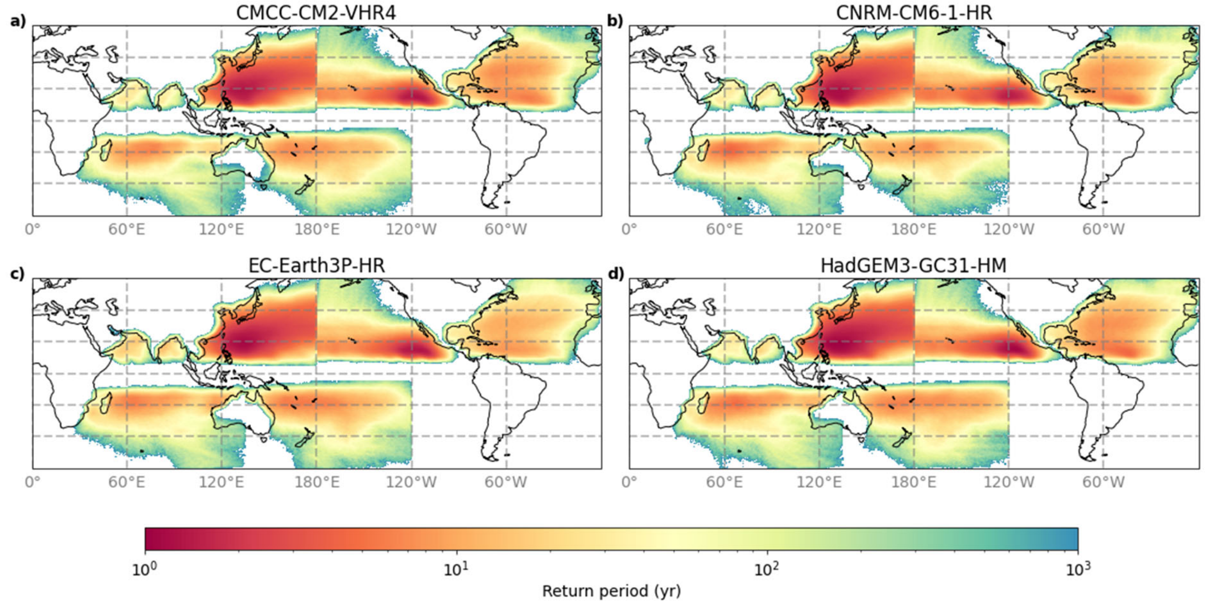

Fig. S10 Spatial distribution of return periods (yr) at 10 km resolution, derived from applying a 2D-wind parameterization to the synthetic tropical cyclone tracks in the STORM+GCM datasets. The return periods are the average value of 1,000 random realizations of 10,000 years of data (sampled with replacement) and determining RPs using Weibull's plotting formula to each realization at Category 1 (maximum 10-minute 10-meter wind speeds ≥ 29 m/s) tropical cyclone strength. Sub-panels show outcomes for each of the four global climate models.

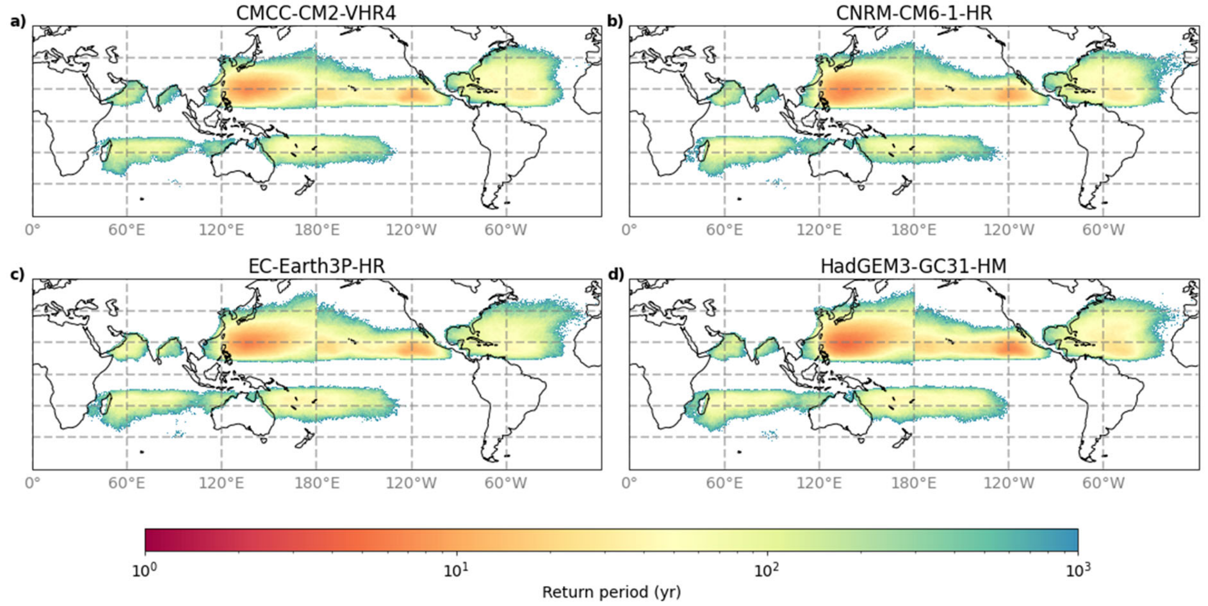

Fig. S11 Same as Fig. S10, but now for Category-3 (maximum 10-minute 10-meter wind speeds ≥ 43.4 m/s) tropical cyclone strength.

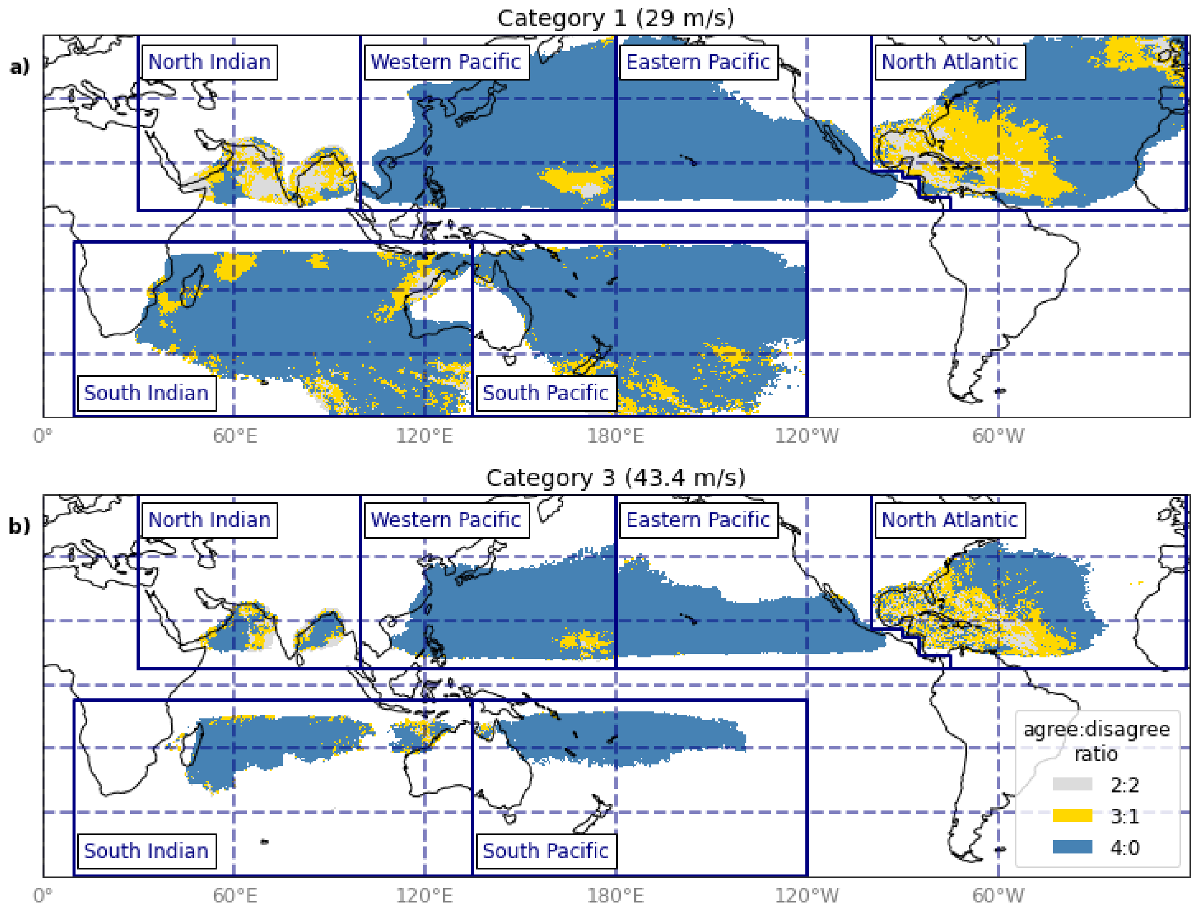

Fig. S12 Number of STORM climate change datasets (one per global climate model; four in total) agreeing on the sign of change compared to the STORM baseline climate datasets.

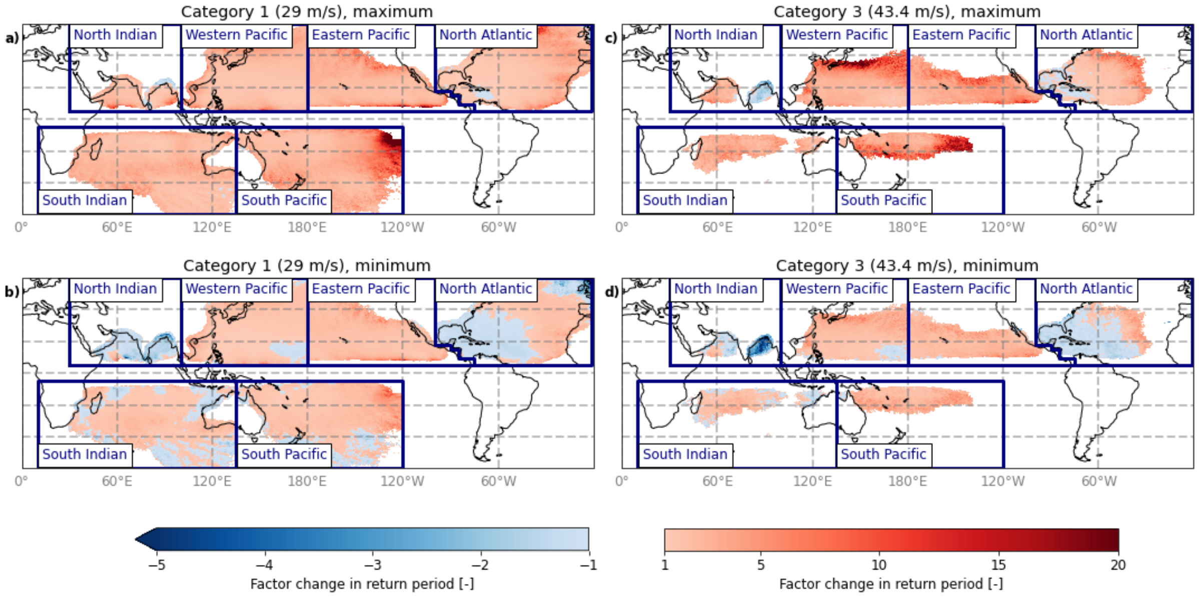

Fig. S13 Maximum (top row) and minimum (bottom row) factor difference in return periods compared to the STORM baseline climate dataset. A negative factor indicates an increase in return period, equivalent to a decrease in probability. A positive factor indicates a decrease in return period, equivalent to an increase in probability.

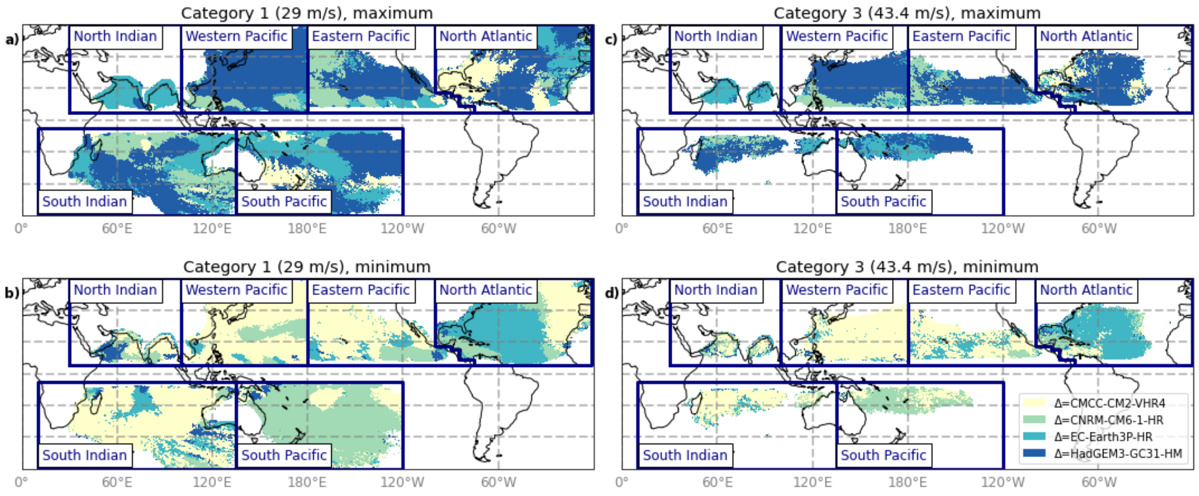

Fig. S14 Global climate model having the highest (top) and lowest (bottom) difference in return period compared to the STORM baseline climate dataset.

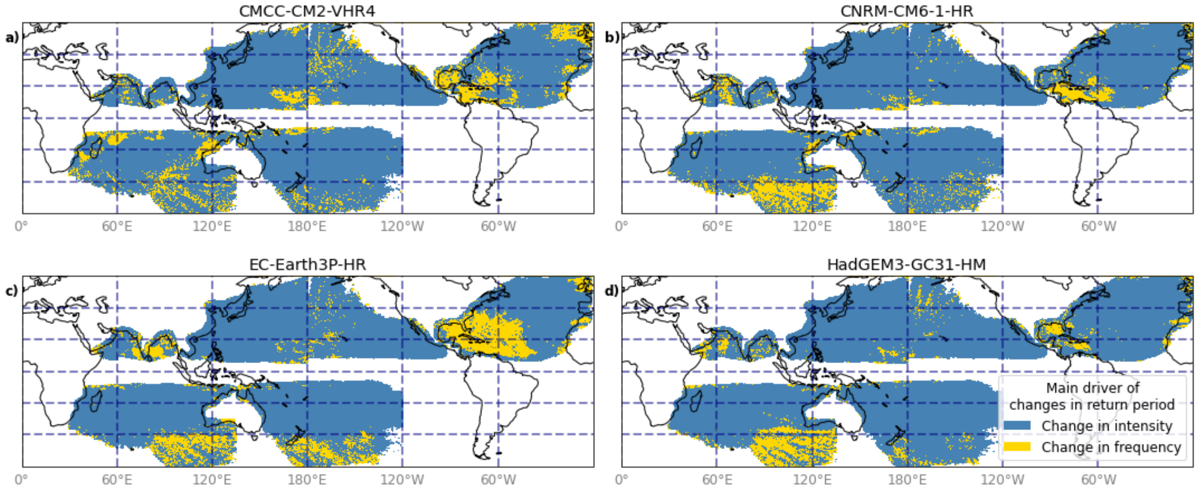

Fig. S15 Main driver of the changes in return period for all global climate models as presented in Fig.4 and Fig. S13 for Category-1 wind speeds. Blue regions represent locations where the change in intensity between the baseline and the future climate is dominant; yellow regions represent locations where the dominant change is a change in frequency.

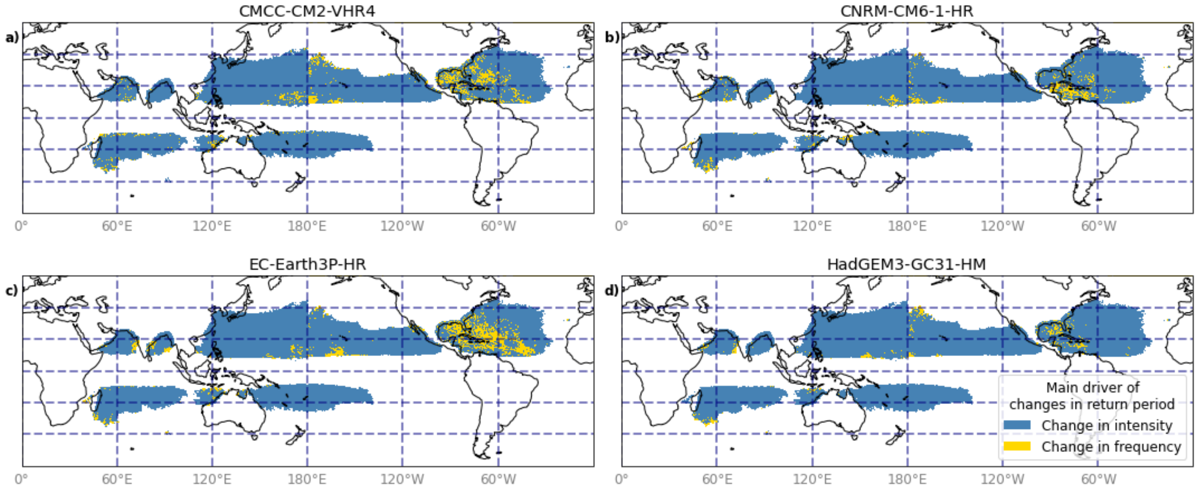

Fig. S16 Same as Fig. S15, but now for Category-3 wind speeds.

Table S8 Basin-wide statistics of the factor change in return period for a Category-1 event between STORM-B (corresponding to the average climate conditions of 1980-2017) and the ensemble median of the STORM-C datasets. These statistics are calculated over the data as visualized in Fig. 4a.

	Eastern Pacific	North Atlantic	North Indian	South Indian	South Pacific	Western Pacific
**Median**	2.42	1.77	1.15	1.81	2.26	1.67
**Mean**	2.82	2.05	1.33	1.93	2.64	2.00
**St. dev.**	1.43	1.08	0.75	0.56	1.41	0.86
**25 and 75% percentile**	1.92 - 3.19	1.21 - 2.62	0.9 - 1.47	1.50 - 2.26	1.78 - 2.96	1.33 - 2.42
**Min**	0.83	0.22	0.27	0.68	0.42	1.02
**Max**	23.62	11.4	10.05	6.16	20.35	9.80

Table S9 Same as Table S8, but now for a Category-3 events (Fig. 4b).

	Eastern Pacific	North Atlantic	North Indian	South Indian	South Pacific	Western Pacific
**Median**	2.98	1.42	1.34	2.48	3.79	2.34
**Mean**	3.31	1.87	1.28	2.57	4.18	3.22
**St. dev.**	1.20	1.12	0.81	0.77	1.78	2.06
**25 and 75% percentile**	2.57 - 3.70	1.12 - 2.21	0.52 - 1.68	2.04 - 2.98	2.74 - 5.31	1.94 - 3.83
**Min**	0.89	0.35	0.21	0.69	0.77	0.86
**Max**	17.13	9.59	6.27	8.05	13.55	21.07

REFERENCES AND NOTES

1. NOAA National Centers for Environmental Information, *U.S. Billion-Dollar Weather and Climate Disasters* (2021); https://www.ncdc.noaa.gov/billions/.

2. T. Knutson, S. J. Camargo, J. C. Chan, K. Emanuel, C.-H. Ho, J. Kossin, M. Mohapatra, M. Satoh, M. Sugi, K. Walsh, Tropical cyclones and climate change assessment: Part II: Projected response to anthropogenic warming. *Bull. Am. Meteorol. Soc.* **101**, E303–E322 (2020).

3. C. M. Patricola, M. F. Wehner, Anthropogenic influences on major tropical cyclone events. *Nature* **563**, 339–346 (2018).

4. N. Lin, K. Emanuel, M. Oppenheimer, E. Vanmarcke, Physically based assessment of hurricane surge threat under climate change. *Nat. Clim. Chang.* **2**, 462–467 (2012).

5. K. Emanuel, Response of global tropical cyclone activity to increasing CO2: Results from downscaling CMIP6 models. *J. Climate* **34**, 57–70 (2021).

6. A. B. Martinez, Forecast accuracy matters for hurricane damage. *Econometrics* **8**, 18 (2020).

7. A. Gettelman, D. N. Bresch, C. C. Chen, J. E. Truesdale, J. T. Bacmeister, Projections of future tropical cyclone damage with a high-resolution global climate model. *Clim. Change* **146**, 575–585 (2018).

8. R. A. Pielke Jr., J. Gratz, C. W. Landsea, D. Collins, M. A. Saunders, R. Musulin, Normalized hurricane damage in the United States: 1900-2005. *Nat. Hazards Rev.* **9**, 29–42 (2008).

9. J. Weinkle, C. Landsea, D. Collins, R. Musulin, R. P. Crompton, P. J. Klotzbach, R. Pielke Jr., Normalized hurricane damage in the continental United States 1900–2017. *Nat. Sustain.* **1**, 808–813 (2018).

10. M. J. Roberts, J. Camp, J. Seddon, P. L. Vidale, K. Hodges, B. Vannière, J. Mecking, R. Haarsma, A. Bellucci, E. Scoccimarro, L.-P. Caron, F. Chauvin, L. Terray, S. Valcke, M.-P. Moine, D. Putrasahan, C. D. Roberts, R. Senan, C. Zarzycki, P. Ullrich, Y. Yamada, R. Mizuta, C. Kodama, D. Fu, Q. Zhang, G. Danabasoglu, N. Rosenbloom, H. Wang, L. Wu, Projected future changes in tropical cyclones using the CMIP6 HighResMIP multimodel ensemble. *Geophys. Res. Lett.* **47**, e2020GL088662 (2020).

11. T. Fiedler, A. J. Pitman, K. Mackenzie, N. Wood, C. Jakob, S. E. Perkins-Kirkpatrick, Business risk and the emergence of climate analytics. *Nat. Clim. Chang.* **11**, 87–94 (2021).

12. H. Murakami, M. Sugi, Effect of model resolution on tropical cyclone climate projections. *SOLA* **6**, 73–76 (2010).

13. R. J. Haarsma, M. Roberts, P. L. Vidale, C. A. Senior, A. Bellucci, Q. Bao, P. Chang, S. Corti, N. S. Fučkar, V. Guemas, J. von Hardenberg, W. Hazeleger, C. Kodama, T. Koenigk, L. R. Leung, J. Lu, J. J. Luo, J. Mao, M. S. Mizielinski, R. Mizuta, P. Nobre, M. Satoh, E.

Scoccimarro, T. Semmler, J. Small, J. S. von Storch, High resolution model intercomparison project (HighResMIP) for CMIP6. *Geosci. Model Dev.* **2016**, 1–35 (2016).

14. C. A. Davis, Resolving tropical cyclone intensity in models. *Geophys. Res. Lett.* **45**, 2082–2087 (2018).

15. J. Chen, Z. Wang, C.-Y. Tam, N.-C. Lau, D.-S. D. Lau, H.-Y. Mok, Impacts of climate change on tropical cyclones and induced storm surges in the Pearl River Delta region using pseudo-global-warming method. *Sci. Rep.* **10**, 1965–1965 (2020).

16. E. D. Gutmann, R. M. Rasmussen, C. Liu, K. Ikeda, C. L. Bruyere, J. M. Done, L. Garrè, P. Friis-Hansen, V. Veldore, Changes in hurricanes from a 13-yr convection-permitting pseudo–global warming simulation. *J. Climate* **31**, 3643–3657 (2018).

17. P. Jyoteeshkumar Reddy, D. Sriram, S. S. Gunthe, C. Balaji, Impact of climate change on intense Bay of Bengal tropical cyclones of the post-monsoon season: A pseudo global warming approach. *Climate Dynam.* **56**, 2855–2879 (2021).

18. C.-Y. Lee, S. J. Camargo, A. H. Sobel, M. K. Tippett, Statistical-dynamical downscaling projections of tropical cyclone activity in a warming climate: Two diverging genesis scenarios. *J. Climate* **33**, 4815–4834 (2020).

19. N. Bloemendaal, I. D. Haigh, H. de Moel, S. Muis, R. J. Haarsma, J. C. J. H. Aerts, Generation of a global synthetic tropical cyclone hazard dataset using STORM. *Sci. Data* **7**, 40 (2020).

20. C.-Y. Lee, M. K. Tippett, A. H. Sobel, S. J. Camargo, An environmentally forced tropical cyclone hazard model. *J. Adv. Model. Earth Syst.* **10**, 223–241 (2018).

21. K. Emanuel, S. Ravela, E. Vivant, C. Risi, A statistical deterministic approach to hurricane risk assessment. *Bull. Am. Meteorol. Soc.* **87**, 299–314 (2006).

22. I. D. Haigh, L. R. MacPherson, M. S. Mason, E. M. S. Wijeratne, C. B. Pattiaratchi, R. P. Crompton, S. George, Estimating present day extreme water level exceedance probabilities around the coastline of Australia: Tropical cyclone-induced storm surges. *Clim. Dyn.* **42**, 139–157 (2014).

23. N. Bloemendaal, H. de Moel, S. Muis, I. D. Haigh, J. C. J. H. Aerts, Estimation of global tropical cyclone wind speed probabilities using the STORM dataset. *Sci. Data* **7**, 377 (2020).

24. K. R. Knapp, M. C. Kruk, D. H. Levinson, H. J. Diamond, C. J. Neumann, The international best track archive for climate stewardship (IBTrACS): Unifying tropical cyclone data. *Bull. Am. Meteorol. Soc.* **91**, 363–376 (2010).

25. G. J. Holland, An analytic model of the wind and pressure profiles in hurricanes. *Mon. Weather Rev.* **108**, 1212–1218 (1980).

26. R. H. Simpson, H. Saffir, The hurricane disaster—Potential scale. *Weatherwise* **27**, 169–186 (1974).

27. K. J. E. Walsh, S. J. Camargo, T. R. Knutson, J. Kossin, T. C. Lee, H. Murakami, C. Patricola, Tropical cyclones and climate change. *Trop. Cyclone Res. Rev.* **8**, 240–250 (2019).

28. K. Bhatia, G. Vecchi, H. Murakami, S. Underwood, J. Kossin, Projected response of tropical cyclone intensity and intensification in a global climate model. *J. Climate* **31**, 8281–8303 (2018).

29. K. Emanuel, Environmental factors affecting tropical cyclone power dissipation. *J. Climate* **20**, 5497–5509 (2007).

30. J. M. Done, M. Ge, G. J. Holland, I. Dima-West, S. Phibbs, G. R. Saville, Y. Wang, Modelling global tropical cyclone wind footprints. *Nat. Hazards Earth Syst. Sci.* **20**, 567–580 (2020).

31. J. P. Kossin, K. A. Emanuel, G. A. Vecchi, The poleward migration of the location of tropical cyclone maximum intensity. *Nature* **509**, 349–352 (2014).

32. J. P. Kossin, K. A. Emanuel, S. J. Camargo, Past and projected changes in Western North Pacific tropical cyclone exposure. *J. Climate* **29**, 5725–5739 (2016).

33. A. Mamalakis, J. T. Randerson, J.-Y. Yu, M. S. Pritchard, G. Magnusdottir, P. Smyth, P. A. Levine, S. Yu, E. Foufoula-Georgiou, Zonally contrasting shifts of the tropical rain belt in response to climate change. *Nat. Clim. Chang.* **11**, 143–151 (2021).

34. C. Bruyère, R. Rasmussen, E. Gutmann, J. Done, M. Tye, A. Jaye, A. Prein, P. Mooney, M. Ge, S. Fredrick, Impact of climate change on Gulf of Mexico hurricanes. *NCAR Tech Note. NCAR/TN535* (2017).

35. United Nations, *About Small Island Developing States* (2021); www.un.org/ohrlls/content/about-small-island-developing-states.

36. United Nations, World Economic Situation and Prospects (2020).

37. B. Tellman, J. A. Sullivan, C. Kuhn, A. J. Kettner, C. S. Doyle, G. R. Brakenridge, T. A. Erickson, D. A. Slayback, Satellite imaging reveals increased proportion of population exposed to floods. *Nature* **596**, 80–86 (2021).

38. B. Neumann, A. T. Vafeidis, J. Zimmermann, R. J. Nicholls, Future coastal population growth and exposure to sea-level rise and coastal flooding–A global assessment. *PLOS ONE* **10**, e0118571 (2015).

39. M. J. Roberts, J. Camp, J. Seddon, P. L. Vidale, K. Hodges, B. Vanniere, J. Mecking, R. Haarsma, A. Bellucci, E. Scoccimarro, L.-P. Caron, F. Chauvin, L. Terray, S. Valcke, M.-P. Moine, D. Putrasahan, C. Roberts, R. Senan, C. Zarzycki, P. Ullrich, Impact of model resolution on tropical cyclone simulation using the HighResMIP-PRIMAVERA multimodel ensemble. *J. Climate* **33**, 2557–2583 (2020).

40. V. Eyring, S. Bony, G. A. Meehl, C. A. Senior, B. Stevens, R. J. Stouffer, K. E. Taylor, Overview of the Coupled Model Intercomparison Project Phase 6 (CMIP6) experimental design and organization. *Geosci. Model Dev.* **9**, 1937–1958 (2016).

41. B. C. O'Neill, C. Tebaldi, D. P. van Vuuren, V. Eyring, P. Friedlingstein, G. Hurtt, R. Knutti, E. Kriegler, J. F. Lamarque, J. Lowe, G. A. Meehl, R. Moss, K. Riahi, B. M. Sanderson, The Scenario Model Intercomparison Project (ScenarioMIP) for CMIP6. *Geosci. Model Dev.* **9**, 3461–3482 (2016).

42. D. P. van Vuuren, J. Edmonds, M. Kainuma, K. Riahi, A. Thomson, K. Hibbard, G. C. Hurtt, T. Kram, V. Krey, J.-F. Lamarque, T. Masui, M. Meinshausen, N. Nakicenovic, S. J. Smith, S. K. Rose, The representative concentration pathways: An overview. *Clim. Change* **109**, 5–31 (2011).

43. IPCC, Climate Change 2021: The Physical Science Basis. Contribution of Working Group I to the Sixth Assessment Report of the Intergovernmental Panel on Climate Change (2021).

44. C. D. Roberts, R. Senan, F. Molteni, S. Boussetta, M. Mayer, S. P. Keeley, Climate model configurations of the ECMWF Integrated Forecasting System (ECMWF-IFS cycle 43r1) for HighResMIP. *Geosci. Model Dev.* **11**, 3681–3712 (2018).

45. O. Gutjahr, D. Putrasahan, K. Lohmann, J. H. Jungclaus, J.-S. von Storch, N. Brüggemann, H. Haak, A. Stössel, Max Planck Institute Earth System Model (MPI-ESM1.2) for the High-Resolution Model Intercomparison Project (HighResMIP). *Geosci. Model Dev.* **12**, 3241–3281 (2019).

46. K. Hodges, A. Cobb, P. L. Vidale, How well are tropical cyclones represented in reanalysis datasets? *J. Climate* **30**, 5243–5264 (2017).

47. P. A. Ullrich, C. M. Zarzycki, TempestExtremes: A framework for scale-insensitive pointwise feature tracking on unstructured grids. *Geosci. Model Dev.* **10**, 1069–1090 (2017).

48. M. J. Roberts, *CMIP6 HighResMIP: Tropical storm tracks as calculated by the TRACK algorithm* (2019); https://catalogue.ceda.ac.uk/uuid/0b42715a7a804290afa9b7e31f5d7753.

49. E. Scoccimarro, A. Bellucci, D. Peano, *CMCC CMCC-CM2-VHR4 model output prepared for CMIP6 HighResMIP* (2017); 10.22033/ESGF/CMIP6.1367.

50. A. Voldoire, *CNRM-CERFACS CNRM-CM6–1-HR model output prepared for CMIP6 HighResMIP* (2019); 10.22033/ESGF/CMIP6.1387.

51. EC-Earth Consortium, *EC-Earth-Consortium EC-Earth3P-HR model output prepared for CMIP6 HighResMIP* (2018); 10.22033/ESGF/CMIP6.2323.

52. M. Roberts, *MOHC HadGEM3-GC31-HM model output prepared for CMIP6 HighResMIP* (2017); 10.22033/ESGF/CMIP6.446.

53. M. K. James, L. B. Mason, Synthetic tropical cyclone database. *J. Waterw. Port. C Div.* **131**, 181–192 (2005).

54. B. Harper, *Tropical Cyclone Parameter Estimation in the Australian Region: Wind-Pressure Relationships and Related Issues for Engineering Planning and Design–A Discussion Paper* (2002).

55. J. Altman, O. N. Ukhvatkina, A. M. Omelko, M. Macek, T. Plener, V. Pejcha, T. Cerny, P. Petrik, M. Srutek, J.-S. Song, A. A. Zhmerenetsky, A. S. Vozmishcheva, P. V. Krestov, T. Y. Petrenko, K. Treydte, J. Dolezal, Poleward migration of the destructive effects of tropical cyclones during the 20th century. *Proc. Natl. Acad. Sci. U.S.A.* **115**, 11543–11548 (2018).

56. S. Sharmila, K. J. E. Walsh, Recent poleward shift of tropical cyclone formation linked to Hadley cell expansion. *Nat. Clim. Chang.* **8**, 730–736 (2018).

57. J. P. Kossin, A global slowdown of tropical-cyclone translation speed. *Nature* **558**, 104–107 (2018).

58. N. Lin, D. Chavas, On hurricane parametric wind and applications in storm surge modeling. *J. Geophys. Res. Atmos.* **117**, D09120 (2012).

59. L. Makkonen, Plotting positions in extreme value analysis. *J. Appl. Meteorol. Climatol.* **45**, 334–340 (2006).

60. B. Jones, B. C. O'Neill, *Global One-Eighth Degree Population Base Year and Projection Grids Based on the Shared Socioeconomic Pathways, Revision 01* (2020); https://doi.org/10.7927/m30p-j498.

61. K. J. E. Walsh, M. Fiorino, C. Landsea, K. L. McInnes, Objectively determined resolution-dependent threshold criteria for the detection of tropical cyclones in climate models and reanalyses. *J. Climate* **20**, 2307–2314 (2007).

62. N. Bloemendaal, I. D. Haigh, H. De Moel, S. Muis, R. J. Haarsma, J. C. J. H. Aerts, *STORM IBTrACS present climate synthetic tropical cyclone tracks*, 4TU.ResearchData (2019); https://doi.org/10.4121/uuid:82c1dc0d-5485-43d8-901a-ce7f26cda35d.

63. N. Bloemendaal, H. De Moel, S. Muis, I. D. Haigh, J. C. J. H. Aerts, *STORM tropical cyclone wind speed return periods*, 4TU.ResearchData (2020); https://doi.org/10.4121/uuid:779b9dfd-b0ff-4531-8833-aaa9c0cf6b5a.

64. N. Bloemendaal, H. De Moel, A. B. Martinez, S. Muis, I. D. Haigh, K. Van der Wiel, R. Haarsma, P. J. Ward, M. Roberts, J. C. M. Dullaart, J. C. J. H. Aerts, *STORM climate change synthetic tropical cyclone tracks*, 4TU.ResearchData (2021); https://doi.org/10.4121/14237678.v1.

65. N. Bloemendaal, H. De Moel, A. B. Martinez, S. Muis, I. D. Haigh, K. Van der Wiel, R. Haarsma, P. J. Ward, M. Roberts, J. C. M. Dullaart, J. C. J. H. Aerts, *STORM climate change tropical cyclone wind speed return periods*, 4TU.ResearchData (2021); https://doi.org/10.4121/14510817.v1.

66. N. Bloemendaal, H. De Moel, A. B. Martinez, S. Muis, I. D. Haigh, K. Van der Wiel, R. Haarsma, P. J. Ward, M. Roberts, J. C. M. Dullaart, J. C. J. H. Aerts, *STORM + STORM future climate scripts* (2022); https://doi.org/10.5281/zenodo.6337644.

67. M. Bister, K. A. Emanuel, Low frequency variability of tropical cyclone potential intensity 1. Interannual to interdecadal variability. *J. Geophys. Res. Atmos.* **107**, ACL 26-21–ACL 26-15 (2002).
